# Magnesium Alloys in Orthopedics: A Systematic Review on Approaches, Coatings and Strategies to Improve Biocompatibility, Osteogenic Properties and Osteointegration Capabilities

**DOI:** 10.3390/ijms25010282

**Published:** 2023-12-24

**Authors:** Gianluca Giavaresi, Daniele Bellavia, Angela De Luca, Viviana Costa, Lavinia Raimondi, Aurora Cordaro, Maria Sartori, Silvio Terrando, Angelo Toscano, Giovanni Pignatti, Milena Fini

**Affiliations:** 1 Scienze e Tecnologie Chirurgiche, IRCCS Istituto Ortopedico Rizzoli, Via di Barbiano, 1/10, 40136 Bologna, Italy; daniele.bellavia@ior.it (D.B.); angela.deluca@ior.it (A.D.L.); viviana.costa@ior.it (V.C.); lavinia.raimondi@ior.it (L.R.); aurora.cordaro@ior.it (A.C.); maria.sartori@ior.it (M.S.); 2 Ortopedia Generale, IRCCS Istituto Ortopedico Rizzoli, Via di Barbiano, 1/10, 40136 Bologna, Italy; silvio.terrando@ior.it (S.T.); angelo.toscano@ior.it (A.T.); giovanni.pignatti@ior.it (G.P.); 3 Direzione Scientifica, IRCCS Istituto Ortopedico Rizzoli, Via di Barbiano, 1/10, 40136 Bologna, Italy; milena.fini@ior.it

**Keywords:** magnesium, alloy, osseointegration, animal models, in vitro models

## Abstract

There is increasing interest in using magnesium (Mg) alloy orthopedic devices because of their mechanical properties and bioresorption potential. Concerns related to their rapid degradation have been issued by developing biodegradable micro- and nanostructured coatings to enhance corrosion resistance and limit the release of hydrogen during degradation. This systematic review based on four databases (PubMed^®^, Embase, Web of Science™ and ScienceDirect^®^) aims to present state-of-the-art strategies, approaches and materials used to address the critical factors currently impeding the utilization of Mg alloy devices. Forty studies were selected according to PRISMA guidelines and specific PECO criteria. Risk of bias assessment was conducted using OHAT and SYRCLE tools for in vitro and in vivo studies, respectively. Despite limitations associated with identified bias, the review provides a comprehensive analysis of preclinical in vitro and in vivo studies focused on manufacturing and application of Mg alloys in orthopedics. This attests to the continuous evolution of research related to Mg alloy modifications (e.g., AZ91, LAE442 and WE43) and micro- and nanocoatings (e.g., MAO and MgF2), which are developed to improve the degradation rate required for long-term mechanical resistance to loading and excellent osseointegration with bone tissue, thereby promoting functional bone regeneration. Further research is required to deeply verify the safety and efficacy of Mg alloys.

## 1. Introduction

Titanium, cobalt–chromium-based alloys and stainless steels are widely used in orthopedics for fixation devices and joint prostheses due to their favorable mechanical properties, corrosion resistance and ability to biologically integrate with the human body (biocompatibility) [1]. Recently, bioresorbable fixation devices have been proposed for certain surgical procedures, such as osteotomies of small bone segments or at the epiphysis level, even in long bones like hallux valgus. These devices do not interfere with X-ray imaging techniques and eliminate the need for a second surgery, which could pose potential risks to the patient and additional costs [2,3].

There is considerable interest in the new generation of orthopedic devices manufactured from magnesium (Mg) and its alloys. Mg and its alloys exhibit favorable biocompatibility and mechanical properties. Indeed, Mg possesses several advantageous mechanical properties: a Young’s modulus of approximately 41–45 GPa, a considerable range of elongation from 3% to 21.8% and an ultimate tensile strength ranging from 160 to 263 MPa. These values are notably close to those exhibited by bone, differentiating Mg from other materials commonly used in orthopedics. With a high specific strength (strength-to-weight ratio of about 130 kN m/kg) and good energy absorption capability, even in load-bearing applications, Mg emerges as a promising candidate in orthopedics. These characteristics contribute to reducing the occurrence of stress shielding phenomena in various procedures [4]. Additionally, its density, ranging from 1.74 to 1.84 g/cm^3^ depending on alloying composition, is comparable to that of bone (1.8–2.1 g/cm^3^), endowing Mg with a lightweight quality. [5]. Although there are concerns about the biocompatibility of Mg-based alloys due to their rapid degradation, it is important to note that the degradation products, including non-toxic Mg ions, promote bone formation, angiogenesis and stimulate bone healing [6]. Magnesium is a cofactor for many enzymes in human metabolism and is also involved in tissue-healing; of note, extra Mg^2+^ do not cause cytotoxicity in the human body and are eliminated through urine [7]. Nevertheless, rapid corrosion in aqueous environments, caused by a low standard electrode potential (−2.37 V), can lead to negative surgical outcomes, such as bone fractures and the release of hydrogen gas. This gas can form bubbles around the implant, accumulating and expanding, thereby impairing the stability of the local microenvironment. Displacement or rupture of the peri-implant tissues may occur, as well as compression of peripheral vessels, leading to cellular damage, pH alteration and, in the worst case, necrosis and bone erosion. From a mechanical standpoint, a too-fast degradation process can compromise the favorable characteristics of the Mg alloy: swift corrosion will harm the mechanical integrity of the Mg implants, resulting in the rapid release of the aforementioned products (hydrogen and hydroxide ions). Consequently, it is imperative to control the degradation rate of Mg implants to ensure an appropriate spatiotemporal complementarity between bone regeneration/remodeling and Mg implant degradation, thereby avoiding compromising the structural integrity. This approach can prevent potential harm to patient health and reduce overall healthcare costs [8].

The use of coatings represents a strategy to improve corrosion resistance and reduce hydrogen release during the degradation of magnesium alloys. Thanks to the advent of the nanotechnologies, nanostructured coatings can also be obtained, further improving the protective barrier function, to limit the contact between the metal surface and environmental elements such as oxygen, water and other chemicals, thereby preventing corrosion. Additionally, coatings can act as a sacrificial layer, allowing the metal to corrode on top of the coating—which can help to slow down the corrosion process and decrease the amount of hydrogen emitted. Coatings can function as a hydrogen diffusion barrier, diminishing blistering, cracking and other damage resulting from the excessive release of hydrogen during Mg alloy degradation. Biodegradable surface coatings can significantly impede rapid degradation and enhance the biological activity of magnesium alloys. Coatings are typically applied to magnesium alloys through mechanical, physical, chemical or biological techniques. The coatings can be divided into metallic coatings such as metal oxides, ceramic coatings or polymeric coatings consisting of both synthetic and natural polymers [9,10].

The objectives of this systematic review, prepared following the PRISMA guidelines [11], were to evaluate the latest approaches, coating materials and strategies proposed up to 2022 for Mg alloys, to address bias risks in the analysis of collected individual studies and to identify the most effective strategies to overcome issues hindering the use of Mg-alloy-based devices. The study commenced with an evaluation of the literature on medical devices for orthopedic applications, specifically examining Mg-based alloys and their possible use in orthopedics. The research focused on the factors that improve osseointegration and probed viable solutions to mitigate the corrosion of these alloys.

## 2. Materials and Methods

### 2.1. Eligibility Criteria

Eligibility criteria defined by the PECO (population, exposure, comparison, outcome) statement were used to select the studies included in the current systematic review. The population of interest (P) was differentiated according to the experimental study models considered: preclinical models, in vivo and in vitro, concerning the use of magnesium alloys for the realization of orthopedic devices. Preclinical in vitro studies included experimental models using human or animal cell lines (except non-mammalian) or primary cells, such as mesenchymal stem cells and osteoblasts, used in monolayer cultures, co-cultures and three-dimensional culture models. For in vivo studies, the following models were included: rodents (e.g., rats and mice of any strain), lagomorphs, sheep and any other experimental animal, without restriction as to sex, age, species, in which such alloys have been surgically implanted in bone to study their osseointegration. The exposure of interest (E) will be magnesium-alloy-based devices, coated or uncoated, or with functionalized surfaces or any other surface modification that improves the osseointegration of such devices. The comparator (C) will be any animal or cell treatment group not exposed to magnesium-alloy-based devices or any cell treatment group exposed to vehicle control only or any animal or cell treatment group exposed to a control alloy already in clinical use, such as a titanium alloy (e.g., Ti6Al4V), which has also been the reference group in clinical trials. Studies without a reference group were excluded. As for the population, the outcomes (O) were also differentiated according to the type of model considered. For in vitro models, the primary outcome was cell adhesion, proliferation/viability and bone matrix deposition and the secondary outcomes were an increase in gene expression (e.g., PCR) and/or specific protein synthesis (e.g., ELISA) related to the osseointegration process. For in vivo models, the primary outcome was the histological appearance of osseointegration of the implanted device and the secondary outcomes were increases in bone histomorphometric parameters such as bone-to-implant contact.

### 2.2. Search Strategy

The bibliographic search was conducted in four databases according the indications of the PRISMA guidelines [11] in the period between 2012 and 2022. All references identified by the search were uploaded to the Rayyan Systematics Reviews research tool (https://www.rayyan.ai/) and duplicate references, narrative and/or systematic reviews, editorial conference abstracts and non-English references were eliminated. The collected references were then assessed for relevance to the current review topic by reading the full text and irrelevant references to population (e.g., animal cell lines and/or primary mesenchymal stem cells and/or osteoblasts), exposure (e.g., magnesium alloy) or outcome (e.g., cell viability, histomorphometric parameters) were excluded. An evaluation of the bibliographic references of the selected papers was also carried out to assess whether further articles could be considered related to the topic of the current review and, if necessary, added manually.

**Figure 1:**
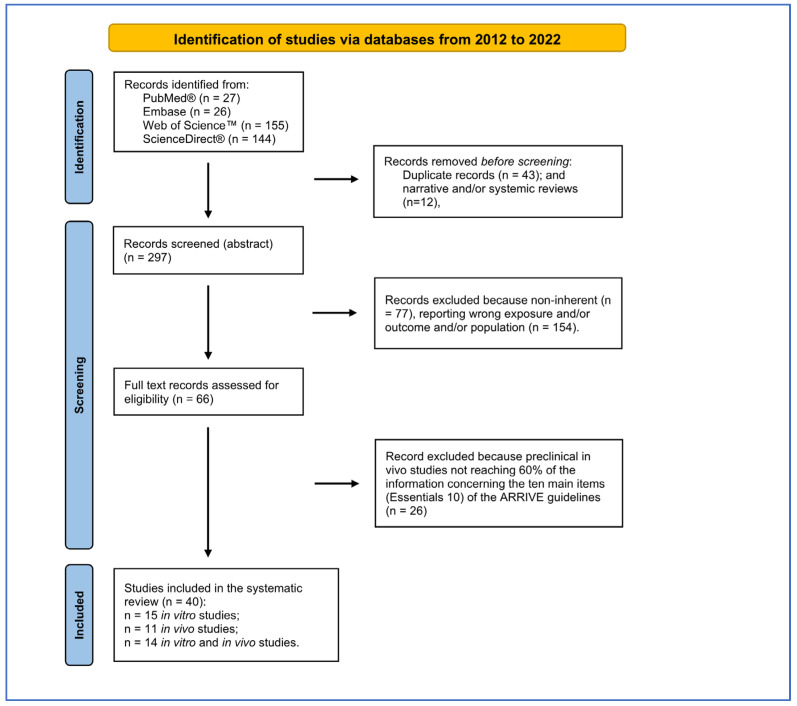
PRISMA flow diagram followed for reference selection process.

This evaluation/selection activity was carried out independently by four authors (D.B., A.D.L., V.C., L.R.) and any uncertainties in the choice of references were resolved by joint evaluation with the intervention of a supervisor (M.F., G.G.). The summary flowchart of the entire reference selection process is reported in Figure 1. Subsequently, two authors (D.B., V.C.) performed a further selection of the selected references of the in vivo preclinical studies, considering those references that reported at least 60% of the information on the ten essential points (Essentials 10) of the ARRIVE guidelines (Animal Research: Reporting of In Vivo Experiments guide) [12] that should be included in a publication reporting the results of animal research. These guidelines ensure that studies are reported in sufficient detail to allow adequate evaluation of the research from a methodological point of view and to allow possible replication of the methods or results. Finally, the selected studies were imported into Zotero (v. 6.0.19), which was used as the reference manager.

#### 2.2.1. Databases

The following databases were used for the literature search:

PubMed^®^—https://pubmed.ncbi.nlm.nih.gov/ (accessed on 12 December 2022)
Embase—https://www.embase.com/ (accessed on 13 December 2022)
Web of Science™—https://access.clarivate.com/ (accessed on 12 December 2022)
ScienceDirect^®^—https://www.sciencedirect.com/ (accessed on 14 December 2022)

#### 2.2.2. Search Strings

The PubMed^®^ search string used a combination of MeSH keywords and Boolean operators (AND/OR/NOT) based on parameters defined in the PECO instruction’s Exposure and Outcome sections. This string was adapted and calibrated to work on Embase, Web of Knowledge and ScienceDirect. Additional supporting information on the search string structure is available in Supplementary File SA.

### 2.3. Parameters Extracted from the Studies

The data extraction method was defined by three reviewers (G.G., D.B., L.R.) to obtain all the information necessary for (Table 1): (1) the risk of bias assessment for in vitro studies (Cochrane RoB2 and OHAT) [13,14]; (2) the evaluation of risk of bias for in vivo studies (SYRCLE) [15] and (3) to report results [14].

### 2.4. Risk of Bias Assessments within Individual Studies

The collected references’ internal quality was assessed by two reviewer groups using risk of bias (RoB) evaluation: one for in vitro studies (A.C., V.C., A.D.L. and L.R.) and the other for in vivo studies (D.B., V.C. and G.G.). Each group conducted an independent assessment. If differences in assessments arose, these were resolved through collegial discussion or with the intervention of a supervisor (M.F. or G.G.) if no agreement was achieved. There is presently no validated tool for assessing the internal validity of in vitro studies. The tools used to date, including Cochrane RoB2 and OHAT, are based on those created for either human or animal studies and cover similar areas. Therefore, to assess the RoB of the in vitro studies, we used OHAT’s tool, which comprises a set of evaluation criteria that are common to each test’s experimental flow. These criteria target the main domains of bias, including selection bias, performance bias, attrition bias, detection bias, selective reporting bias and other sources of bias. For each domain, five alternative responses were available for each question. “Definitely Low Risk of Bias”, “Probably Low Risk of Bias”, “Probably High Risk of Bias”, “Definitely High Risk of Bias” or “Not Reported”. Regarding the assessment of the RoB in in vivo preclinical studies, we utilized the SYRCLE RoB tool, which shares the same bias domains as the OHAT tool. The SYRCLE RoB tool provides three possible answers: “Low risk of bias”, “Unclear risk of bias” and “High risk of bias”.

## 3. Results

The bibliographic search led to the selection of 352 records from four search engines (Figure 1). After 43 duplicates and 12 reviews were removed, the remaining 297 records were screened using Rayyan software. The PRISMA checklist is reported as Supplementary File SB.

Two-hundred-thirty-one records were excluded because they did not correspond to the eligibility criteria defined by PECO, reported incorrect populations, exposures or outcomes or were narrative reviews. Of the 66 records assessed for eligibility, 26 records were excluded because they did not reach 60% of information required by the ten main elements (Essentials 10) of the ARRIVE guidelines. Of the forty records selected, fifteen concerned in vitro studies, eleven were in vivo studies and fourteen were in vitro and in vivo (nine were considered only for the in vitro part because the in vivo part did not reach 60% of the Essential 10 of ARRIVE). The list of items extracted from in vitro and in vivo studies have been in the Supplementary File SC.

### 3.1. Risk of Bias Assessment

**Figure 2:**
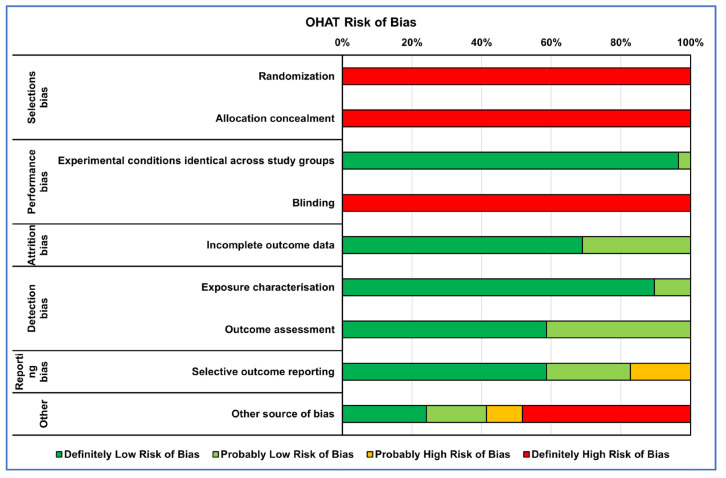
Heatmap of percentage OHAT RoB for in vitro studies.

**Figure 3:**
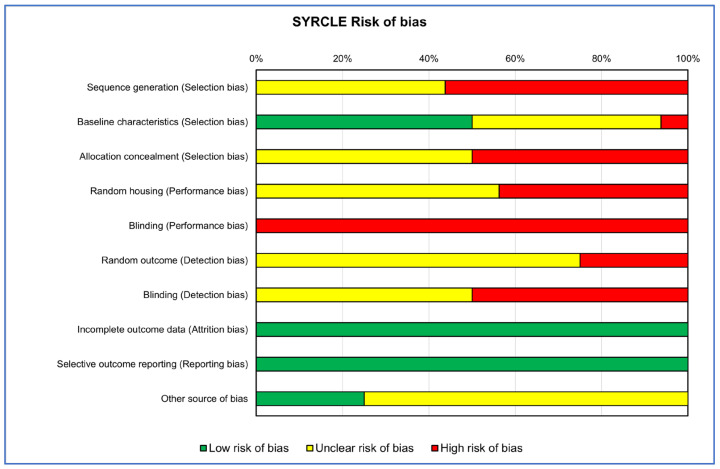
Heatmap of percentage SYRCLE RoB for in vivo studies.

The RoB evaluation of the studies reporting both in vitro and in vivo data was performed using OHAT and SYRCLE tools for the in vitro and in vivo parts, respectively, receiving those studies’ two RoB evaluations. RoB summaries are presented through heatmaps (Figure 2 and Figure 3). The selection bias mainly concerned the methods of assigning the experimental models to the various treatments, which did not occur in random assignment or were not described, leading precisely to the risk of selection of the best models for certain treatments, influencing the experimental results (high risk of bias in vitro: 100%; in vivo: 38%). Also, for the performance bias, both the method of assignment and the researchers’ knowledge of the treatments assigned to specific models could have led to the risk of overestimating the performance of a treatment (high risk of bias in vitro: 50%; in vivo: 72%). As regards the detection bias of the in vivo studies, the analysis conducted on the selected literature highlighted how this was mainly determined by the lack of a methodology for analyzing the blinded results or the assignment of cases to be analyzed always being random (high risk of bias in vivo: 38%).

Finally, for both types of study, the results of ‘Other sources of bias’, counted for in vitro studies as ‘Definitely high risk’ for 45% and for those in vivo as ‘Unclear risk of bias’ for 65%, were due to not clearly reporting which software or statistical test was used for the analysis and, if present, often not suitable for that type of data, as well as not reporting a priori power analysis, especially for in vivo studies, and the significance level adopted.

### 3.2. Narrative Results Synthesis

#### 3.2.1. In Vitro Studies

The high degradation rate of Mg alloys represents one of the major issues to handle to improve their biocompatibility and osseointegration capability. The results of the literature review indicate that efforts to modify the properties of magnesium (Mg) can be classified into three main lines of action, which are often interconnected: (i) the development of alloys that combine Mg with key elements; (ii) the exploration of novel surface treatment techniques, also able to create and/or incorporate nanostructures, aimed at controlling Mg degradation, improving biocompatibility and enhancing interactions with cells and tissues involved in the healing process (e.g., micro-arc oxidation, MAO) and (iii) the exploiting of organic/inorganic nanocomposites, which play a crucial role in bone metabolism due to their biomimetic and structural properties, thereby promoting cell growth. The synthesis of in vitro results follows these three main lines of action. Table 1 reports the in vitro studies selected on the osseointegration capability of Mg-based alloys, uncoated (mainly control groups) or coated with various functionalized surfaces, ranging from inorganic to more complex treatment until nutraceuticals.

##### In Vitro Studies of Mg Alloys without Coatings

In vitro studies provide compelling evidence demonstrating that functionalizing Mg with elements of various natures (e.g., germanium, zirconium, rare elements like gadolinium, silver, strontium, etc.) significantly enhances the performance of the material. These improvements manifest in terms of enhanced cellular viability, improved adhesion characteristics and increased osteogenic differentiation capability of the investigated cell types [16,17,18].

##### In Vitro Studies of Mg Alloys with Inorganic Coatings

In addition to the creation of Mg alloys with the different elements, positive results were also obtained through the exploration of different innovative surface modification techniques. Surface modification is an effective way of altering the biological performance of an implant device. Surface properties, including hydrophilicity, roughness and chemical composition, play important roles in cellular and bacterial responses.

For example, micro-arc oxidation (MAO), an electrochemical surface approach to generate a microporous and adherent coating of alloy, was used by Liu et al., who added lithium (Li) to AZ91 alloy (Li-MAO) to improve the angiogenic and osteogenic activity of Mg, obtaining a nanoporous coating [19]. The direct and indirect interaction of rat bone mesenchymal stromal cells (rBMSCs) with MAO, Li-MAO and AZ91 samples showed that rBMSCs spread better in MAO and Li-MAO surfaces in comparison with AZ91 control substrate, affected by corrosion and wider corrosive gaps. Li-MAO improved the proliferation of cells, the expression of high levels of genes connected to the osteogenic differentiation as runt-domain transcription factors 2 (Runx-2), alkaline phosphatase (Alp), collagen type I alpha 1 chain (Col1a1) and osteocalcin (Ocn) and an increase in mineralization nodule formation compared to AZ91 alloy. Through the Western blot assay, Liu et al. hypothesized that the nanoporous Li coating positively influenced osteogenic differentiation by activating the Wnt/β-catenin pathway. The authors carried out other investigations that showed no differences in the corrosion resistance between MAO and Li-MAO, which was in any case superior to that of AZ91, and which highlighted the better angiogenic capacity of Li-MAO.

The efficiency of the MAO approach was also investigated by Shangguan et al., who compared the biological effects of calcium-phosphate (Ca-P)-contained MAO coating, pulse electrodeposition (PED) Ca-P coating and strontium phosphate (SrP) conversion coating in MC3T3-E1 cell line [20]. A cell viability assay revealed that cells cultured with extracts derived from Ca-P MAO increased their proliferation rate, and, after 7 days of treatment, high levels of ALP protein release, especially in the Ca-P MAO group, followed by the Sr-P coating, were observed compared to other samples. Following the possibility of using Mg alloy as a delivery of soluble factors to improve its osteointegration attitude, Kim et al. modified AZ31 alloy through layer-by-layer coating (LBL), the MAO approach and hydrothermal treatment (HT) for 24 h with Bone Morphogenetic Protein (BMP)-2 at various concentrations (20, 50 or 100 ng/mL). These biomaterials were tested indirectly for proliferation and osteogenic abilities on MC3T3 cell lines [21]. The data suggested a strongly positive effect of BMP50 and 100 coated to AZ31 on cell adhesion ability and ALP expression compared to other samples.

Xie et al. used the plasma electrolytic oxidation (PEO) technique to modify the surface of the Mg alloy, fabricating through simple immersion processes a construct of manganese (Mn) and iron (Fe) oxyhydroxide duplex layers on the PEO-treated AZ31 (PEO-Mn/Fe) [22]. Through this combined approach, the authors deeply explored the possibility of using rare earth elements to improve corrosion resistance and limit the release of ions. The C3H10T1/2 cells were seeded onto the scaffolds, and bioactivity data revealed that the PEO-Fe/Mn scaffold promotes cell growth, alkaline phosphatase activity and bone-related gene expression after 3–7 days of treatment. Furthermore, Li et al. tested biofunctional zinc (Zn) and Fe co-decorated Mg-based implants with nanoflower morphology using zinc-doped ferric oxyhydroxide nanolayer Mg alloy (PEO-FeZn, PEO-Fe and PEO-Zn) [23]. The results demonstrated that direct contact of PEO-Fe and PEO-FeZn1 and PEO-Zn2 on C3H10T1/2 cell line induced an increase in cell proliferation, ALP release and mRNA expression of Runx2, Al, Ocn and Opn during the experimental times. Meanwhile, the mineralization efficiency evaluated by Alizarin Red S (ARS) assay showed that the PEO-FeZn2 group displayed an increase in mineralization nodules formation compared to other samples. Li et al. investigated the possible use of hydroxyapatite (HA) as an outer layer of a ceramic coating of Mg alloy combining PEO and hydrothermal treatment, validating the osteoinductive role of a new bilayer-structured coating (termed HAT). This coating comprises an outer layer of HA nanorods and an inner layer of pores-sealed MgO with HA/Mg(OH)_2_ [24]. Viability assessment on Rabbit Mesenchymal Stromal Cells (RBMSCs) and human fetal osteoblast cell line (hFOB) 1.19 seeded on the HAT-coated Mg alloys revealed an increase in proliferation, as well as bone sialoprotein and osteopontin secretion and formation of mineralization nodules. Meanwhile, the mineralization efficiency evaluated by Alizarin Red S assay showed that the PEO-FeZn_2_ group displayed an increase in mineralization nodules formation compared to other samples.

Another way to enhance the corrosion resistance and cytocompatibility of AZ31 scaffolds is the use of fluoride treatment to acquire an MgF2 coating. Yu W et al. revealed that the MgF2-coated AZ31 (FAZ31) scaffolds enhanced proliferation, attachment and osteogenic ability of rBMSCs maintained on FAZ31 more than on AZ31 scaffolds [25]. Another biomimetic compound to increase Mg scaffold osteointegration and biocompatibility is fluoridated hydroxyapatite (FHA). FHA coating possesses a nanoneedle structure that can mimic collagen fibers and is derived from hydroxyapatite (HA), where OH- in the HA lattice is substituted with F- to form FHA. FHA nanocoatings, due to their optimal biocompatibility, biodegradability and osteogenic properties, can be easily coated onto biodegradable Mg substrates by electrochemical deposition because they are more stable, with low dissolution and high cell response. Shen et al. demonstrated the biological effects of direct contact of AZ31 alloy coated with a biomimetic FHA and HA via a microwave aqueous approach with MC3T3-E1 cell line. The data revealed that FHA and HA coatings promoted a reduction in cell proliferation and an increase in osteogenic differentiation, as confirmed by reverse transcriptase-polymerase chain reaction (RT-PCR) analysis and ALP staining [26] The role of FHA was also described by Cao Z. et al., who investigated the bioactivity role of FHA and tantalum (Ta) on Mg samples in MC3T3 cell lines [27]. The obtained data suggested that the combination of the nanoneedle structure and Ta ion’s function would synergistically enhance the osteogenic properties and cell proliferation of Mg/FHA and Mg/FHA/Ta groups more than on another sample.

Plasma immersion ion implantation (PIII) was used to realize a functionalized titanium oxide (TiO_2_) in TiO_2_/Mg_2_TiO_4_ nanolayer on the surface of WE43 magnesium by Lin Z et al., 2019 [28]. Adopting this approach, the investigated cells displayed an increase in cell viability and an induction of ALP protein release, osteogenic markers expression and mineralization nodules deposition after 14–21 days of treatment with the TiO_2_/Mg_2_TiO_4_ nanolayer compared to the WE43 control alloy.

Cheng et al. evaluated the effects of pure Mg alloy uncoated or coated with layered double hydroxides (LDH) (Mg-Al LDH and Mg(OH)_2_) on MC3T3-E1 maintained with derived extract alloys [29]. The cells treated with Mg-Al LDH samples displayed significantly higher cell proliferation, osteointegration abilities and mineralization nodules deposition amount compared to other samples. These data were also confirmed by Cheng et al., who investigated nanostructures of AZ31 Mg alloy treated with a Mg−Al layered double hydroxide (AZ31 Al-LDH) with the same cell line model [30].

Zhang et al. presented evidence of the effect of osteogenic markers expression after co-treatments with Ca–P-coated, Sr–P-coated and uncoated Mg–Sr alloy discs, immersed in different pH grading solutions, on an RMSCs model [31]. After 7 and 21 days of culture, RMSCs on Sr–P coating showed an increase in cell proliferation, ALP release and mineralization nodules formation compared to the other groups. The same effect on proliferation and osteogenic differentiation was observed in MC3T3-E1 cell line treated with extracts derived by AZ91-3Ca Mg alloy modified using diammonium hydrogen phosphate and calcium nitrate (both named CP) to obtain CP4100 and CP710 by Ali et al. It was demonstrated that CP4100 and CP710 induce an increase in proliferation, cells adhesion and osteogenic markers expression of Runx-2, Col-1a and Alp compared to AZ91-3 Ca Mg alloy [32].

Regarding the third line of action, hydroxyapatite (HA), the most important inorganic component of human bones, was investigated as coating for Mg also doped with Sr at different concentrations in comparison to uncoated ZK60 Mg alloy. The rBMSCs maintained on the surface of the alloys showed an increase in proliferation ability and osteogenic genes expression, as well as ALP protein release, in Sr-doped HA-coated samples compared to the HA-coated alloys [33]. In order to improve the stability, compact structure and efficiency of the osteointegration process of HA coatings on Mg alloys, You et al. developed a series of HA-based coatings through a hydrothermal treatment of brushite precursor (DCPD) [34]. MC3T3-E1 cell lines seeded on Mg DCPD revealed an increase in cell viability, mitochondrial activity, cell adhesion and osteogenic differentiation.

The electrospinning process was used by Perumal et al. to realize a cylindrical mesh cage implant with circular holes of AZ31 magnesium coated with a nanocomposite material containing polycaprolactone (PCL) at different percentages, pluronic F127 and nanohydroxyapatite (nHA), which significantly improve viability, osteogenic activity and mineralization activity of MG63 in comparison to AZ31 alloy alone [35].

##### In Vitro Studies of Mg Alloys with Organic Coatings

The effects of AZ31 Mg alloy coated with corrosion-resistant silane enriched with high- and low-molecular-weight hyaluronic acid (HA) (hHA-AZ31 and l-HA-AZ31) were tested in correlation with titanium alloy coated in the same way by Agarwal et al. Biocompatibility tests revealed that HA-Ti had the ability to increase cell proliferation, adhesion and osteogenic gene expression [36].

Another compound useful for medical applications is dexamethasone (Dex), a type of corticosteroid well-known to facilitate osseointegration. Lee J.H. et al. investigated the effects of direct contact of Mg coated with Dex/Black phosphorus (BP)/poly(lactide-co-glycolide)(PLGA) with the MC3T3 cell line. The results highlighted a strong proliferation and osteointegration ability of Mg-Dex/BP/PLGA compared to the control alloy [37].

Peng et al. fabricated through hydrothermal film a Zn-contained polydopamine (PDA) film to coat AZ31 in order to enhance osteogenic abilities and also improve antibacterial and anti-inflammatory action [38]. The extracts derived by these alloys were used to evaluate some main biological parameters like proliferation ability and adhesion, which were enhanced by coatings in comparison to the control alloy for MC3T3-E1 cell line. qRT-PCR analysis of osteogenic genes and ALP protein release on cells highlighted no differences in the expression of Runx-2 and collagen type II A (Coll II A) between the different coatings. Moreover, the extract of the Zn-contained PDA-coated sample was able to activate RAW264.7 polarization to the M2 phenotype (anti-inflammatory type) compared to the control alloy, suggesting anabolic activity also from an immunological point of view.

The osteogenic role of extracts derived from AZ31 Mg alloy coated with HA or HA/chitosan–metformin (HA/CS-MF), through hydrothermal treatment, on MC3T3-E1 cells was also investigated by Li et al. [39]. The osteogenic data revealed a significant increase in the expression of osteogenic genes and ALP release in the AZ31/HA/CS-MF extract, indicating that the AZ31/HA/CS-MF had the strongest osteogenic induction ability compared to AZ31 and AZ31/HA. This was further confirmed by the results of the viability tests analysis.

Li et al. investigated the osteoimmunomodulation effects of curcumin WE43 Mg-coated alloy on reducing inflammation around an implant and favoring its osseointegration [40]. They fabricated a three-layered coating on Mg with a different combination of polylactic acid (PLA) containing curcumin-loaded F-encapsulated mesoporous silica nanocontainers (cFMSNs) in different amounts and dicalcium phosphate dehydrate (DCPD). The higher cFMSN content exhibited better corrosion protection of the Mg substrate at 21 days of immersion and the presence of curcumin induced an increase in ALP release, osteogenic genes expression and mineralization nodules deposition. Furthermore, curcumin release induced a rapid macrophage phenotype change from M1 to M2, significantly downregulating pro-inflammatory factors (TNF-α, IL-1β, iNOS) and upregulating anti-inflammatory cytokines (IL-10, CD206, ARG), resulting in a higher immunomodulatory efficiency.

The role of Mg alloy on osteoclast activation and immunomodulation was investigated by Negrescu et al. [41]. The electrospinning technique was used to fabricate poly(ε-caprolactone) (PCL) fibers loaded with coumarin (CM) and/or zinc oxide nanoparticles (ZnO) using the commercial AZ31 Mg alloy as single and combined formulas. The results obtained on RAW 264.7 cells treated with extracts of alloys revealed an increase in viable cells and an induction of macrophage–osteoclast differentiation, as shown by tartrate-resistant acid phosphatase (TRAP) staining and actin cytoskeleton staining of the receptor activator of nuclear factor kappa-B ligand (RANKL)-stimulated RAW 264.7 cells.

Concerning this advantage, Cheon et al. investigated a new biomimetic alloy in which Ta was deposited onto the surface of a poly(ether imide) PEI coating on magnesium implants using a plasma ion immersion implantation (PIII) technique [42]. The Ta/PEI-coated Mg induced an increase in cell viability and ALP activity in the MC3T3-E1 cell line compared to PEI-Mg coated after 10 days of direct contact with them.

A recent investigation suggested the role of hyaluronic acid associated to berberine (HA/BBR) as a component of a new biomaterial with antibacterial properties. Zhang et al. tested the effects of this new biomaterial on MC3T3-E1 cell lines [43]. Cell viability and osteogenic analysis demonstrated that this innovative materials combination showed excellent cell compatibility and induced an increase in ALP release and calcium nodules deposition compared to other samples.

Pandele et al. investigated another biological component used as a biomimetic scaffold—resveratrol, which was covalently immobilized onto cellulose acetate polymeric membranes used as a coating on a Mg-1Ca-0.2Mn-0.6Zr alloy, named Mg-CA-Res [44]. The Mg-CA-Res induced a significant increase in cell proliferation (MC3T3 cell line), ALP activity and extracellular matrix mineralization after 7 and 14 days of treatment compared to Mg-CA.

#### 3.2.2. In Vivo Studies

In vivo studies have shown that combining Mg alloy with organic/inorganic molecules is the most widely studied method for enhancing material properties. Thirteen selected in vivo studies evaluated the osseointegration capabilities of uncoated or coated Mg alloy with different types of inorganic and/or organic substances. Table 2 provides a summary of information from selected in vivo studies ordered by the biomaterial type investigated: Mg alloys without coating, those with simple or successive inorganic coatings and finally Mg alloys coated with organic molecules. The primary objective of all the included in vivo studies is to assess the degradation and/or resorption profile of the different materials and evaluate their replacement capabilities with newly formed bone.

##### In Vivo Studies with Mg Alloys without Coatings

Kleer et al. compared two different Mg alloys: La2 and LAE442 in comparison to porous ß-tricalcium phosphate scaffolds [45]. The gas formation evaluated with radiological examination indicates a similar distribution of gas accumulations in the musculature in the two Mg alloys and no gas formation in controls. The microtomography analyses showed that LA2 alloy was degrading faster and in a dis-homogeneous manner compared to the LAE442 alloy, which exhibited better osseointegration, suggesting its possible examination in weight-bearing bone defects [45].

Using the synchrotron (SRμCT), Krüger et al. assessed the degradation and osseointegration of Mg alloys combined with gadolinium (Gd) [8]. The authors implanted Mg-5Gd and Mg-10Gd screws in comparison with Ti screw and polyether-ether-ketone (PEEK), demonstrating better osseointegration and newly formed bone in degraded Mg alloy zones compared to the other two materials, where the bone results were more mature. However, Mg-10Gd was preferred compared to Mg-5Gd as it degraded faster and less homogeneously than Mg-10Gd [8].

A Mg-based biodegradable alloy with germanium (Ge) was developed by Bian et al. [16]. The selected Mg-3 Ge alloy showed good performance in terms of resorption with high resistance to degradation compared to other alloys and optimal osseointegration with the bone that grows onto the surface of the implant, with a possible complete reabsorption in three months [16].

Lindtner et al. showed that a biodegradable magnesium alloy based on WE43 (Mg-Y-Nd-HRE composition) has several advantages compared to implants that use other degradable implant materials, such as reinforced PLGA [46]. The study showed that the WE43 had a significantly greater BV/TV compared to the self-reinforced PLGA implants taken as a control in the early analysis (4 weeks of implantation). However, there were no significant differences observed at other times (12 and 24 weeks). Importantly, push-out testing revealed highly significantly greater shear strength (τu) in the Mg alloy in all implantation periods. Although it showed clear signs of local degradation, osseointegration was not impaired, as indicated by newly formed bone that covered the surface of degraded implant parts with the same degree of unaltered parts, without noticeable local or systemic inflammation. Furthermore, the WE43 alloy exhibited markedly superior bone–implant interface strength and a greater amount of peri-implant bone than the self-reinforced copolymeric controls [46].

Liu et al., investigated magnesium alloy with scandium (Sc), one of the rare earth elements, ref. [47] used in its β-phase, which had been reported to exhibit a shape memory effect (Mg-30% wt Sc alloy). With respect to animal studies, this alloy exhibited slow degradation, with an ions Sc release far from the chronic toxicity level of this element. Only a little gas generation was observed without bone homeostasis perturbation at the initial stage compared to the HP-Mg used as control, wherein the released gas influenced bone remodeling. However, a problem of this alloy is the possible excess presence of impurities (especially Fe, Ni and Cu) that must be controlled as this leads to too rapid degradation of the Mg alloy, resulting in an excessive release of Sc ions [47].

##### In Vivo Studies with Mg Alloys with Inorganic Coatings

Kopp et al. showed the long-term (18-month) osseointegration abilities of the Mg-Ca-Zn (ZX00MEO)-based Mg alloy screws when the surface was modified through plasma-electrolytic oxidation (PEO) compared to non-surface-modified screws [48]. Implanted screws showed improved absorption behavior through reduced degradation rates, faster bone formation and increased quality around the modified implants [48].

PEO techniques were also used for surface modification of the WE43-based locking plates and screws by Rendenbach et al. to evaluate osteosynthesis and osseointegration at 6 and 12 months [49]. Regarding radiological and histological screw and plate degradation, PEO-treated WE-43 implants showed high wear resistance, ensuring a good seal of the implant, as high degradation can lead to the failure of the fixation. Furthermore, the decrease in degradation also determined a low hydrogen gas release, reducing the associated risk of perturbation of bone healing. The bone–implant contact percentage (BIC) and lamellar bone quantification evidenced how PEO surface modification had beneficial effects, improving osseointegration. In fact, PEO surface modification of WE43 screws and plates had no impact on the surrounding bone quality in comparison to untreated implants, although the surrounding BV/TV was reduced in both implants when compared with unimplanted animals. However, no control with animals with a screw hole without an implant was performed, so it remains unclear if this effect was due to the biomaterial or the surgical procedure itself [49]. Xie et al. used a simple immersion process to construct Mn and Fe oxyhydroxide duplex layers on the PEO-treated AZ31 (PEO–Mn/Fe) implant, which showed the best corrosion resistance compared to other unmodified and modified alloys (AZ31, PEO AZ31, PEO-Mn AZ31, PEO-Fe AZ31) [22]. Furthermore, a rat femur implantation experiment showed that the PEO–Fe/Mn–coated AZ31 alloy had the best bone regeneration and osteointegration abilities. In fact, large voids between the formed bone and the implants were observed in the PEO and PEO–Mn groups, while the PEO–Mn/Fe group had increased bone formation compared to other implant groups and the bone adhered more closely to the implant surface, indicating it to be the most favorable for bone regeneration and osteointegration [22].

Witting et al., starting from their previous studies on LAE442 alloy in porous form, evaluated the effectiveness of coatings of magnesium fluoride (MF2), combined with polylactic acid (PLA) or Ca-P, in slowing the degradation of the LAE442 alloy in promoting osseointegration in a rabbit model, using TCP as a control [50]. Although the coating of MF2 improved the rapid degradation with gas formation of this alloy, it did not control it. The results relating to osseointegration indicated that the presence of Ca-P compared to the other coatings increased bone regeneration in terms of bone volume and newly formed trabeculae. Conversely, as regards the speed of degradation, PLA slowed down this process but did not control the strong gas production, which was better controlled by the Ca-P coating [50].

In the study of Cheng et al., where Zr and N ions were simultaneously added into AZ91 Mg alloys by plasma immersion ion implantation (PIII), it was observed that there was a change in implant volume and bone formation around the modified implants relative to simple AZ91 implants, which were used as the control [17]. It was shown that the modified implant volume decreased much more slowly, and there was a corresponding increase in the amount of newly formed bone relative to the controls. In addition to the increase in the amount of bone that formed on the implant surface, obvious gas evolution was not observed in the Zr-N-implanted AZ91 group, in contrast to the AZ91 implants, which had adverse effects on cell adhesion, growth and differentiation [17].

Yu et al. have used a fluoride treatment to acquire a MgF2 coating with better corrosion resistance compared to other coatings [25]. This coating had discrete biocompatibility and an effective corrosion protective layer, which enable abundant new bone formation in the defects, which grew into the pores of the scaffolds. An obvious resorption zone with void formation was observed around the AZ31 scaffolds, whereas the FAZ31 surface constituted a stable and biocompatible interface for the attachment of osteoprogenitor cells, as well as subsequent proliferation, differentiation, calcification and final bone formation [25].

A functionally modified TiO_2_/Mg_2_TiO_4_ nanolayer on WE43 implant, through the plasma ion immersion implantation (PIII) technique, was investigated by Lin et al. [28]. The increase in new bone formation adjacent to the treated WE43 was higher than that of the titanium control and untreated WE43. Furthermore, this layer seemed to exhibit photoactive bacterial disinfection ability when irradiated by ultraviolet light due to intracellular reactive oxygen species (ROS) production, indicating how the TiO_2_/Mg_2_TiO_4_ nanolayer in these implants can significantly promote new bone formation, suppress bacterial infection and contain the degradation of implants simultaneously. The obtained results also evidenced that not only did PIII-treated WE43 samples stimulate new bone formation significantly compared to untreated implants but they also restored the mechanical property of the formed bone similarly to the level of the surrounding mature bone.

##### In Vivo Studies with Mg Alloys with Organic Coatings

Finally, Li et al. investigated the role of a self-healing coating with a three-layered structure and containing curcumin at different concentrations, described above, in favoring osseointegration and modulating the inflammatory response to Mg alloy degradation [40]. Their multifunctional coatings showed high corrosion resistance four weeks after implantation, and those with the highest curcumin concentration (20FMSN) modulated its surrounding immune microenvironment towards anti-inflammation (downregulation of TNF-α, IL-1β and iNOS, as well as an upregulation of IL-10, CD206 and ARG), thus facilitating osseointegration compared to the coatings with moderate (10FMSN) and lowest (5FMSN) amounts of curcumin [40].

## 4. Discussion

In recent years, there has been a growing interest in devices based on magnesium alloys, with a focus on biocompatibility features, mechanical properties, performance and strategies developed or adopted to overcome the degradation drawback. This interest is clearly demonstrated by numerous studies, encompassing both experimental approaches and narrative as well as systematic reviews [4,51,52]. However, preclinical studies on these alloys still exhibit various biases, as highlighted in the current review through a risk analysis of the selected papers. In vitro studies were found to be particularly problematic due to selection bias, while in vivo studies were affected by performance bias, both of which can compromise internal validity. Selection bias is often caused by an inadequate or poorly described randomization process or non-concealed allocation, leading to an overestimation or underestimation of the exposure effect and resulting in inaccurate conclusions about the effectiveness of using these alloys. Furthermore, researchers’ awareness of the groups to which the assessed outcomes belong determines the latter, leading to unequal assessment of outcomes between groups and possible biased results. Additionally, more than 60% of the identified high risk of bias or probably/unclear risk of bias in the ‘other sources of bias’ category were attributed to the lack of consistent reporting of statistical information.

In general, the major critical aspect of Mg alloys is represented by its rapid degradation, which results in hydrogen formation in peri-implant tissues and consequent impairment of bone regeneration and limitation in the osseointegration process. To overcome this drawback, several types of alloy modifications have been proposed with the aim of ensuring more homogeneous and controllable degradation phenomena.

None of the modifications proposed in the studies selected for the review reported relief of post-implantation clinical complications in bone tissue. Mg alloys La2 and LAE442 showed slow and homogeneous degradation, more efficient in the LAE442 alloy with retention in device shape throughout the experimental time and providing a better osseointegration [45]. The strategy of adding specific elements like Ag or Gd to the Mg alloy did not lead to satisfactory results in terms of better control of degradation, both in in vitro and in vivo evaluations and in the cellular response to high doses of released Mg ions [18], although the results of osseointegration were interesting [8]. The addition of germanium (>2.5 wt% Ge) to achieve an increasingly better mechanical performance and biodegradability of the Mg alloy provided an improvement in corrosion resistance and a contained production of hydrogen at the implant site that did not affect its osseointegration [16]. The Mg-Y-Nd-HRE alloy, based on the WE43 one, also demonstrated superior osseointegrative capabilities compared to a polymeric control hypothesized for the fixation of small bone lesions [46]. Finally, the modification of WE43 alloy with the addition of Sc (Mg-30 wt%Sc) showed improvement in corrosion rate while still maintaining mechanical properties suitable for bone [47].

Another alternative used to control excessive corrosion of Mg alloys and improve their osseointegration is surface modification with overlay coatings. Literature findings, summarized in the present review, suggest how nanotechnologies serve as a highly valuable tool for modifying and improving the surfaces of Mg alloys. Nanoflowers, nanorods and nanoneedles represent some of the morphologies that can be adopted to modify Mg alloys’ surfaces by using MAO, PEO and hydrothermal treatment, MgF2, oxides or a combined scheme of surface modification techniques. These modifications have demonstrated beneficial properties such as bioabsorbable implant materials and have proven their safety and performance at the device level. MAO and PEO are similar processes involving the electrolysis of a conductive material immersed in an electrolyte but differ in terms of plasma generation, process control and the coating structures produced. MAO coatings often contain ceramic phases and may be porous, while PEO coatings are often denser and more uniform than those produced by MAO. In the studies selected in the current review, MAO was used to improve corrosion resistance and biocompatibility [19,20], as in the case of the results obtained for lithium MAO nanocoating [19]. Calcium phosphate MAO coating on Mg-Sr alloy exhibited the greatest performance in terms of alloy degradation, cell proliferation and alkaline phosphatase activity when compared to Sr-P and Ca-P PED coatings, suggesting it could hold potential for use in orthopedics [20]. Regarding the use of PEO, Kermasorb^®^ demonstrated its biocompatibility and ability to reduce the rate of degradation of ZX00 alloy, which was also confirmed by the presence of residual material up to 18 months after implantation, and improved its osseointegration [48]. The ability of PEO to control the degradation rate was confirmed also for WE43 alloy, which is progressively depleted over the period of 12 months of implantation, resulting in improved osseointegration in part due to its moderate osteostimulative effect [49].

The most interesting results are obtained from multifunctional and composite coatings. PEO-treated Mg alloys’ performance was improved by adding nanobiofunctionalized monolayer or multilayer coatings through immersion or hydrothermal treatments [22,23,24]. The PEO-FeZn nanolayer on AZ31 alloy improved in vitro osteogenic differentiation and showed a specific antibacterial activity by blocking bacterial immune evasion and promoting activation of the NOX-ROS signaling axis of neutrophils [23]. The duplex nanosheet film with an inner layer of MnOOH and an outer layer of FeOOH on PEO-coated AZ31 alloy improved the ability to induce osteogenesis in vitro and bone regeneration and osseointegration in vivo due to the enhanced corrosion resistance and the timed release of bioactive ions (e.g., Mg, Fe and Mn) [22]. The bilayer-structured coating (HAT) composed of an outer layer of hydroxyapatite nanorods and an inner layer of pores-sealed MgO with HA/Mg(OH)_2_, was realized on Mg surface, adopting plasma electrolytic oxidation and hydrothermal treatment. This composite coating is able to modulate anti-inflammatory macrophage response, suppress osteoclastogenesis and facilitated the recruitment and differentiation of osteogenic cells in the surrounding environment [24]. The barrier effect of HAT against body fluids prevented them from reaching the Mg substrate, delaying its degradation and allowing the new bone to act as an additional, more pronounced barrier.

Single-step hydrothermal processing has shown promise in its ability to generate coatings with good adhesion and high crystallinity on rigid substrates. The Ca-P coatings deposited by the single-step hydrothermal method had the effect of reducing the degradation rate of AZ91-3Ca alloy by 60% and hydrogen gas evolution rate by 65% [32]. Additionally, they demonstrated better biocompatibility and viability and increased osteogenic differentiation when compared to pre-osteoblasts, according to in vitro studies. The association of MAO and hydrothermal treatment to immobilize different concentrations of BMP-2 on Ca-P coating improved biocompatibility, delayed the degradation rate of AZ31 and promoted bone formation through sustained BMP-2 release; the BMP-2 concentration was below 50 ng/mL [21].

MgF_2_-based coatings were proposed to coat AZ31 (FAZ31) and LAE442 (PLA- or CaP-P) alloys [25,50]. The latter was also supplemented with the addition of Ca-P or PLA, and there was an important improvement in corrosion resistance, biocompatibility and osteogenic activity in both studies. FAZ31 and Ca-P had superior osteoconductive and osteoinductive properties, increasing the osseointegration process. However, scaffolds with PLA coating degraded more slowly despite increased gas formation. Additionally, fluorinated hydroxyapatite (FHA) was used to coat AZ31 and AZ31B alloys [26,27]. The FHA coating, also in association with hydrothermal synthesis and magnetron sputtering, improved osteogenic differentiation in vitro and offered favorable long-term protection for Mg alloy, increasing the corrosion resistance of the implants [26,27].

Mg alloy coatings with metal nitrides and/or oxide layers, such as the combination of ZrN, ZrO_2_ and Al_2_O_3_ [17], TiO_2_ and Mg_2_TiO_4_ [28], layered double hydroxides (LDH) with Al(NO_3_)_3_ [29,30] or ceramics such as SrP and CaP [31], Sr-doped hydroxyapatite [33] or hierarchically structured hydroxyapatite [34], seem to enhance corrosion resistance by providing a favorable implant surface for cell adhesion, viability and growth, thus improving osteogenesis in vitro, which, in turn, further promotes bone regeneration and osseointegration. Furthermore, a self-antibacterial capability due to oxidative stress induced by ROS production has been highlighted for TiO_2_/Mg_2_TiO_4_ nanolayers [28].

Another interesting way to improve Mg alloy degradation rate, osteogenic and osseointegration potentials is the development of composite coatings [35,36,37,38,39,42]. Most of them enhance in vitro osteoblast differentiation and ECM mineralization, such as the combination of PCL/pluronic/nHA electrospun on AZ31 mesh cage [35], the high-molecular-weight hyaluronic-acid-functionalized silane-coated AZ31 Mg alloys (h-HA-AZ31) [36] or the double coating with osteogenic dexamethasone-loaded black phosphorus and poly(lactide-co-glycolide) (Mg-Dex/BP/PLGA) [37]. In addition, some composite coatings improve macrophages’ polarization into M2 phenotype to reduce inflammation, such as polydopamine films coupled with Zn ions deposited on hydrothermal-treated AZ31 alloy (Mg-Al LDH) [38] or hydroxyapatite/chitosan–metformin (HA/CS-MF) composite coating achieved on AZ31 alloy by hydrothermal treatment [39]. Although tantalum magnetron sputtering onto the surface of poly(ether imide) coatings demonstrates excellent corrosion protection, it does not show any evidence of osseointegration [42].

Finally, the inclusion of natural compounds such as resveratrol, coumarin, curcumin or berberine in coatings brought benefits in relation to their ability to modulate the inflammatory response, always deriving from the degradation of the Mg alloys [40,41,43,44]. The three-layered coating with its self-healing function containing different concentrations of curcumin reduced the rate of Mg alloy degradation and improved osteodifferentiation and osteointegration proportionally to the increasing concentration of curcumin [40]. The macrophage inflammatory activity and osteoclast differentiation response to AZ31 alloy coated with an electrospun composite of PCL fibers loaded with coumarin and/or ZnO nanoparticles were mainly influenced by the ZnO and coumarin presence, providing the best corrosion behavior and the most favorable response in terms of morphological behavior, cell survival and proliferation [41]. The covalent immobilization of resveratrol to CA coating applied to the Mg alloy proved to be effective in vitro in stimulating the vitality and differentiation of osteoblasts and mineralization of the extracellular matrix [44]. Very interesting, and equally complex, is the use of berberine associated with hyaluronic acid to create a multilayer antibacterial coating with a composite based on magnesium hydroxide and poly(alendronate sodium methacrylate)/poly(dimethyldiallylammonium chloride)/poly(ethylene glycol) diacrylate on AZ31 Mg alloy (MgA-Mg(OH)_2_-PALNMA/PDADMAC/PEGDA). The topological structure of the composite pattern seems to be responsible for the high antibacterial capacity and its biocompatibility and osteogenic differentiation [43].

As far as methodology is concerned, the most frequently used cells in the in vitro studies were the Mouse Osteoblastic cell line MC3T3-E1 (62%) [17,18,20,21,26,27,28,29,30,34,37,38,39,42,43,44], followed by Osteosarcoma Cell Line MG63 (7%) [16,35], BMSCs (21%) derived from rat [19,33], rabbit, [24,31] or human [25,40], Mouse Fibroblast-like cell lines (CH3/10T1/2) (7%) [22,23] and Human Fetal Osteoblastic cell line (hFOB 1.19) (3%) [24]. Few studies also investigated the effects of different Mg alloys on the osteoclast cell activation using Mouse Macrophage cell line RAW 264.7 [38,39,40,41]. Most of the collected in vitro studies primarily adopted extracts methodology rather than direct cell-material contact, which imposes limitations on the extent of conclusions that can be drawn from the results. This methodological choice is likely attributed to the release of degradation products from the Mg materials upon contact with cells, which could potentially have adverse effects on cell cultures before the actual effects can be accurately determined. The search strategy did not yield any co-culture studies or advanced in vitro models, which could have introduced a higher level of complexity to the investigations and increased the relevance of the results. All the studies adopted the same tests to evaluate cell behavior in terms of viability (e.g., live and dead, MTT and CKK8), cell adhesion and morphology (e.g., SEM or confocal microscopy) and to understand the osteointegration capability of tested Mg-based alloys, such as qRT-PCR analysis of osteogenic genes, Western blot technique, ELISA assay, Alizarin Red and Sirius red dye staining for cellular and extracellular matrix (ECM) proteins.

For in vivo studies, the most used animal model was the rat (54%) [8,17,22,28,40,46,47], followed by the rabbit (31%) [16,25,45,50]; only two studies were carried out on a large animal model (minipig 15%) [48,49]. Regarding the implant site, the Mg alloy implants were inserted in femoral condyle (38%) [17,25,28,40,47], trochanter (23%) [22,45,50] or diaphysis (15%) [46,49], tibial metaphysis (15%) [8,16] and frontal bone (8%) [48]. In most of in vivo studies, the Mg alloy implants had porous cylinders (31%) [25,45,46,50] or non-porous cylindrical shapes (46%) [8,17,22,28,40,47] or sample screws (15%) [16,48] or plates fixed with screws (8%) [49]. All these studies used microtomography to evaluate in vivo and/or ex vivo degradation of implanted Mg alloys, while osseointegration was assessed through histological evaluation with different staining (e.g., hematoxylin–eosin; Toluidine Blue-Pyronine Y; Lévai–Laczkó, Giemsa, Van Gieson’s and Picrofuchsin). Out of the thirteen in vivo studies, only two of them specifically examined the mechanical competence of the bone tissue regenerated after Mg device implantation. Considering the unique characteristics of these materials, it is crucial to conduct more comprehensive investigations to accurately assess the quality and mechanical competence of the regenerated bone.

Although not an aim of this systematic review, an additional literature search was conducted to determine whether any of the approaches presented here had been evaluated at a higher level, including systematic reviews, meta-analyses and clinical trials. The search retrieved few studies, comprising a single systematic review and four studies (two pilot, one retrospective and one prospective) [2,53,54,55]. At least four of these papers referred to the use of the MgYREZr (magnesium, yttrium, rare earth and zirconium) alloy from Syntellix AG (Hannover, Germany), employed as a screw for the consolidation of comminuted fractures, hallux valgus and tibial osteotomies. Overall, these articles emphasized that the MgYREZr alloy is non-inferior to titanium screws in terms of biocompatibility, resistance to mechanical loading and not requiring removal due to its absorption and not producing artifacts in CT. Only one article referred to the use of Mg-5wt%Ca-1wt%Zn alloy, which demonstrated bone healing and complete resorption one year after treatment. This is attributed to the continuous degradation of the Mg alloy and the potential formation of a biomimetic calcification matrix (CCP) at the degradation interface, which may have contributed to the bone formation process [56].

## 5. Conclusions

This systematic review summarizes 40 preclinical in vitro and in vivo studies on the use of Mg alloys for the manufacturing of fracture fixation devices. The in vitro results indicate three main approaches to modify the properties of magnesium (Mg): (i) developing Mg alloys with key elements, (ii) exploring surface treatment techniques to control degradation and improve biocompatibility and (iii) using organic/inorganic nanocomposites to promote cell growth and mimic bone properties. Furthermore, in vivo studies have demonstrated extensive research on combining Mg alloys with various organic and inorganic molecules to enhance material properties, specifically focusing on osseointegration and modulation of material degradation. In particular, the review reveals the progress in research on Mg alloy modifications (e.g., AZ31, AZ91, LAE442 and WE43) and types of coatings (e.g., PEO, MgF_2_ and oxides) to achieve improved materials in terms of degradation rate. Ensuring long-term mechanical resistance to loading and excellent osseointegration with bone tissue are crucial to promote functional bone regeneration for fracture healing. Nanotechnologies have certainly improved this field of application, and the realization of nanostructures and/or nanocoating has certainly enhanced the characteristics of these peculiar materials by improving their corrosion resistance and osseointegrative properties.

Despite promising advances in Mg alloys, it is essential to emphasize that further investigation is necessary to establish and confirm their efficacy and safety, especially when the nanodimension is involved. Despite the limitations associated with selection, performance and detection bias, the present systematic review offers a broad overview of the development of Mg alloys for orthopedics. These limitations could have been mitigated by implementing rigorous research methodologies or guidelines to ensure reliable and comprehensive results.

## Figures and Tables

**Table 1:** Selected in vitro studies included in the systematic review.

Species, Cell Line/Source, Sex	Mg Alloys Control	Performed Tests, Experimental Time, Replicates and Statistical Analysis	Main Results	Ref.
Human, Osteosarcoma cell lines MG-63	99.95 wt% Mg with different percentage of Ge: Mg-Ge. 99.95 wt% Mg	ALP activity at 5 days. N = 3. One-way ANOVA followed by Tukey test (SPSS 18.0). Type I error was set at 5%.	Higher ALP concentration for Mg-1.5Ge and rolled-Mg-3Ge among the different tested percentages of Ge.	[16]
Mouse, Osteoblastic cell line MC3T3-E1.	AZ91 Mg alloy ingot (Mg with Al 9 wt.% and Zr 1 wt.%): Zr-N- AZ91 Mg. AZ91 Mg alloy.	Live/Dead assay at 1 and 3 days. MTT assay at 1, 3 and 7 days. ALP staining assay at 7, 14 days. N = 4. Student’s *t*-test. Type I error was set at 5%.	Improved cell proliferation and adhesion for Zr-N-implanted Mg in comparison to control AZ91. Increased ALP activity for the Zr-N-implanted AZ91 Mg alloys in comparison to unimplanted AZ91 Mg alloy.	[17]
Mouse, Osteoblastic cell line MC3T3-E1	Mg-1.75 wt%Ag: Mg2Ag. Mg-10.5%Gd: Mg10Gd. Mg 99.99%: Pure Mg.	All investigations were carried out onto corroded (1, 2 or 3 days immersed in DMEM+10%FBS) and non-corroded Mg samples. Live/Dead staining at 24 h. Rhodamine-phalloidin staining of cytoskeleton at 24 h. SEM at 24 h to evaluate MC3T3-E1 adhesion to Mg samples. Immunocytochemistry at 2, 4, 8 and 12 days. Western blot (WB) analysis at 2, 4, 6, 8, 10 and 12 days. N = 3. One-Way ANOVA (SPSS). Type I error was set at 5%.	A time-dependent decrease in viability for MC3T3-E1 cultured on Pure Mg samples pre-corroded; significant viability reduction for MC3T3-E1 cells cultured on pre-corroded Mg2Ag and Mg10Gd. Increase cell extensions (F-actin-based structure) for corroded Mg alloys especially on Pure Mg, Mg2Ag and Mg10Gd pre-corroded for 1 to 2 days. Increase in Runx2 and Collagen I protein expression by WB analysis in non-corroded Mg10Gd for a long time than other samples.	[18]
Rat, Bone marrow mesenchymal stem cells (rBMSCs).	Micro-arc oxidation (MAO) nanoporous coating on AZ91 Mg (MAO) through a 0.04 mol/L NaH_2_PO_4_ and 0.1 mol/L Ca(CH_3_COO)_2_ solution at 450 V for 3 min. Lithium MAO nanoporous coating on AZ91 Mg (Li-MAO) was obtained by immersing MAO samples in 0.02 mol/L LiCl, 0.04 mol/L NaH_2_PO_4_ and 0.1 mol/L Ca(CH_3_COO)_2_ solution at 450 V for 3 min AZ91 Mg alloy.	Cell Morphology and adhesion evaluated by SEM at 3 days. Cell viability analyzed by Live/Dead and Cell counting kit-8 CCK-8 assay at 3 days. ALP Immunofluorescence staining and activity at 7 days. RT-PCR analysis at 7 and 14 days. Alizarin Red Staining at 14 days. One-way ANOVA followed by the Student–Newman–Keuls (SPSS v.20). Type I error was set at 5%.	SEM analysis showed a better morphology and rBMSCs spreading on MAO and on Li-MAO materials groups compared with AZ91 group. Best cells viability for Li-MAO at both day 1 and day 3 in comparison to other materials. Higher percentage of ALP positive cells for Li-MAO group. Highest level of expression of Runx-2, Alp, Col1A1and Ocn genes at both experimental times for. Li-MAO group Higher stained nodules with Alizarin red for Li-MAO group.	[19]
Mouse (C57BL/6), Osteoblastic cell line MC3T3-E1.	Mg-1.5 wt.% Sr (Mg-Sr) alloy coated with Ca-P MAO in the solution 8 g/L KF·2H_2_O, 4 g/L (NaPO_3_)_6_ and 0.8 g/L Ca(OH)_2_, at 20–25 °C, 360 V, 1000 Hz, duty cycle 40% for 5 min; - Ca-P pulse electrodeposition (PED) in the solution 0.042 mol/L Ca(NO_3_)_2_, 0.025 mol/L (NH_4_)H_2_PO_4_ and 0.1 mol/L NaNO_3_, at pH was 4.6, 1 kHz, 30%, 60 °C for 40 min; strontium phosphate (SrP) conversion after immersion in a solution containing 0.1 M Sr (NO_3_)_2_ and 0.06 M NH_4_H_2_PO_4_ at 80◦C for 10 min, with pH adjusted to 3.0 by dilute HNO_3_. Mg-Sr	MTT assay to evaluate cell viability at 1, 3 and 5 days. ALP activity test performed at 3, 5 and 7 days. N = 3 ANOVA followed by Tukey test to test difference among groups (SPSS v.17). Type I error was set at 5%.	Higher viability of MC3T3-E1 on Ca-P MAO coating than on SrP and PED coatings and on Mg-Sr alloy. Highest ALP activity for MC3T3-E1 cells on Ca-P MAO coating, followed by SrP coating, PED coating and Mg-Sr alloy,	[20]
Mouse, Osteoblastic cell line MC3T3-E1.	AZ31 were treated in the following order: Layer by Layer coating (LBL coating = Carrier 1: MAO Coating + Carrier 2: Hydrothermal treatment for 24 h) (A) AZ31 + (L) LBL: MAO coating + Hydrothermal treatment for 24 h: AL. AL coated with BMP-2 at various concentrations (20, 50 or 100 ng/mL), obtaining the following samples: -AL + 20 ng/mL of BMP-2 treatment for 24h: ALB20 -AL + 50 ng/mL of BMP-2 treatment for 24 h: ALB50 -AL + 100 ng/mL of BMP-2 treatment for 24 h: ALB100. AZ31B alloyed (A).	Cell morphological analysis performed at 1, 3 and 5 days. ALP activity at 1 and 2 weeks. N = 3. One-way ANOVA (SPSS v.12). Type I error was set at 5%.	No differences in cell morphology between coatings Increasing in cell nucleuses and cytoplasm for ALB 50 in comparison to other materials at 5 days. Greater ALP expression for ALB groups in comparison to control group.	[21]
Mouse, Fibroblast-like cell lines C3H/10T1/2	Mn and Fe oxyhydroxide duplex layers on the PEO-treated AZ31: PEO-Mn/Fe. AZ31-PEO–Mn. AZ31-PEO–Fe. AZ31 Mg Alloy.	Live/Dead Staining at 3 days. AlamarBlue assay at 1, 3 and 5 days. RT-PCR analysis at 3 and 7 days for Runx-2, Col1A–1, Alp, Ocn and Opn. ALP staining at 3 and 7 days. N = 4 Two-way ANOVA followed by Tukey’s post hoc test (GraphPad Prism 8.3.0.) Type I error was set at 5%.	Low viability for cells with PEO sample surface; higher viability for cells cultured with PEO–Fe and PEO–Mn/Fe samples extract than with the PEO–Mn samples. Increase in Osteogenic gene expression during the experimental times. for cells cultured with PEO–Mn/Fe Higher ALP activity for PEO–Fe and PEO–Mn/Fe groups at both time points.	[22]
Mouse, Fibroblast-like cell lines C3H/10T1/2	AZ31 Mg alloy disks were submitted to a zinc-doped ferric-oxyhydroxide nanolayer-modified PEO coating: -PEO-Fe. -PEO-FeZn1. -PEO-FeZn2. AZ31 Mg alloy.	Live/Dead fluorescent staining assay at 3 days. SEM. Extracellular matrix mineralization (ECM) assay at 14 days. ALP activity assay at 7 and 14 days. RT-PCR at 7 and 14 days. N = 4 Data distribution was tested by Shapiro Wilk test. One-way and two-way ANOVA followed by Tukey’s post hoc test (SPSS 19.0 software). Type I error was set at 5%.	Cell viability results showed that the coatings did not negatively affect the cells after 3 d of co-culture, which were denser and more evenly distributed on the PEO-Fe and PEO-FeZn coatings. Cell morphologies by SEM confirmed the live/dead results. Higher ECM synthesis and ALP staining in each group during experimental times, especially in PEO-FeZn2 group. Highest amount of mineralization calcium nodules for PEO-FeZn2 group compared to PEO-Fe and PEO-Zn1 group. Increased expression of Runx2, Alp, Ocn and Opn gene in PEO-Fe and PEO-FeZn1 groups with increasing incubation duration.	[23]
Rabbit Mesenchymal stromal cells (RMSCs) Human Fetal Osteoblastic cell line (hFOB 1.19) cells	β-glycerophosphate disodium, Ca(OH)_2_,and NaOH dispensed through PEO technique to Mg Alloy: PEO-Mg. PEO-Mg was subjected to hydrothermal treatment (HT): PEO-HT. PEO-HT treated with aqueous solution containing C_10_H_12_CaN_2_Na_2_O_8_ (Ca-EDTA) and NaOH into the autoclave to immerse the PEO-coated Mg and HA at 90 °C for 24 h: HAT/Mg Mg alloy	Fluorescence staining assay of the mineralization. RT-PCR of osteogenic genes and proteins. N = 4. A one-way ANOVA followed by a least-significant-difference post hoc test (SPSS v.16). Type I error was set at 5%.	Increase fluorescent expression of bone sialoprotein (Bsp) and Osteopontin (Opn) for PEO-HT and HAT/Mg Increased expression of ALP, Bsp, Opn and Osteocalcin (Ocn) for HAT/Mg alloy.	[24]
Human, Bone marrow mesenchymal stromal cells (BMSCs)	AZ31 coated with MgF2: FAZ31. AZ31Mg alloy.	Live/Dead staining after 1, 3 and 7 days. SEM and Confocal Laser Scanning Microscope (CLSM) at 7 days of culture. ALP staining assay at 4, 7 and 10 days. RT-PCR analysis of the osteogenesis-related genes (Runx-2, Bmp-2, Ocn, Opn) expression on days 7 and 14. N = 3. One-way ANOVA and Student–Newman–Keuls post hoc tests. Type I error was set at 5%.	Increased cell viability for FAZ31 samples; no cell growth for AZ31 samples. Morphology analysis revealed a 100% confluence and well-spread morphology for cells on FAZ31 samples. Enhanced ALP activity and expression of osteogenesis-related genes for FAZ 31 in comparison to AZ31.	[25]
Mouse, Osteoblastic cell line MC3T3-E1	AZ31 Mg alloy coated with a biomimetic fluoridated hydroxyapatite: FHA. AZ31 Mg alloy coated with hydroxyapatite: HA. AZ31 Mg alloy.	Cell proliferation at 1 day,4 days and 7 days. RT-PCR analysis of Runx2, Ocn and Col1a1at 4 and 7 days ALP staining assay at 4 and 7 days. Independent sample test using SPSS v.19. Type I error was set at 5%.	Reduced cells proliferation and increased cells differentiation for FHA and HA coating in comparison to control. Enhanced osteogenic differentiation and ALP staining for FHA coating significantly.	[26]
Mouse, Osteoblastic cell line MC3T3-E1	AZ31B magnesium coated with fluorinated hydroxyapatite (FHA). AZ31B magnesium coated with tantalum (Ta). AZ31B magnesium coated with FHA and Ta (Mg/FHA/Ta). AZ31B Mg alloy.	Cell morphology analysis by Fluorescence microscopy (FM) methods. MTT assay, ALP staining and Alizarin Red S assay at 4 and 7 days. N = 6. One-way ANOVA using the Fisher’s least-significant-difference multiple comparison test. Type I error was set from 5% to 1%.	Optimal cell spreading and lamellipodia formation, for Mg/FHA and Mg/FHA/Ta groups followed by Mg/Ta and control samples. Higher cell viability for Mg/FHA/Ta at 4 days and 7 days. in comparison to other samples. Higher ALP activity and Alizarin Red S staining for Mg/Ta, Mg/FHA and Mg/FHA/Ta in comparison to Mg pure.	[27]
Mouse, Osteoblastic cell line MC3T3-E1.	WE43 substrates treated by the plasma immersion ion implantation (PIII) technique to obtain TiO_2_/Mg_2_TiO_4_ nanolayer: PIII-treated WE43. WE43 Mg alloy.	Cell adhesion at 5 h. MTT assay at 1 and 3 days. RT-PCR analysis for Col1A-1, Alp, Runx-2, Opn. ALP staining at 3, 7 and 14 days. Alizarin red S assay at 21 days. N = 3. One-way ANOVA (SPSS).	Higher cell adhesion on surface PIII treated WE43 in comparison to WE43 samples. Higher viability on PIII WE43 samples than WE43 control. High expression of osteogenic markers and on PIII WE43 Significantly higher Alizarin red staining for PIII-treated group in comparison to untreated one.	[28]
Mouse, Osteoblastic cell line MC3T3-E1	Mg is covered by: -Mg(OH)_2_ film prepared with 50 mL ultrapure water added with 400 μL NaOH (10 M) was gently pour into the Teflon-line: Mg- Mg(OH)_2_. -Layered double hydroxides (LDH) film obtained with 50 mL Al(NO_3_)_3_ (0.02 M) added with 600 μL NaOH (10 M) was gently pour into the Teflon-line stainless: Mg-LDH. Reaction kettles were kept at 120 °C. After 12 h, the samples were taken out, ultrasonically cleaned for 5 min and rinsed with deionized water. Pure Mg alloy uncoated.	CCK-8 analysis at 1, 3, 5 and 7 days. RT-PCR analysis at 3 and 7 days for Bmp-2, Col1A-1, Alp and Ocn Alizarin Red S assay at 7 days. ALP staining at 3 and 7 days. N = 3. Two-way ANOVA followed by Tukey’s post hoc test (SPSS v.19). Type I error was set at 5%.	Significantly higher cell proliferation for Mg-Mg(OH)_2_ and Mg-LDH samples exhibited. Significantly higher osteogenic expression for Mg-LDH sample Higher osteogenic differentiation for Mg-LDH alloy.	[29]
Mouse, Osteoblastic cell line MC3T3-E1	AZ31 Mg alloy treated with a Mg−Al layered double hydroxide: Mg−Al LDH. AZ31 Mg alloy.	Live/Dead staining at 3 days. SEM at 1, 4 and 24 h. RT-PCR analysis measured at 7 days for Alp, Col1A-1, Opn and Runx-2. ALP staining and Collagen Secretion analysis at 7 days. One-way ANOVA, followed by using Tukey’s post hoc test (SPSS v.19). Type I error was set at 5%.	Comparable viability for cells cultured onto Mg−Al LDH film and on AZ31alloy. Improved cell adhesion for Mg−Al LDH in comparison to AZ31 sample. Higher osteogenic differentiation for Mg−Al LDH-coated alloy in comparison to control.	[30]
Rabbit, Mesenchymal stem cells (RMSCs)	Mg-1.5 wt.% Sr (Mg-Sr) alloys were coated with - CaP (Mg-Ca–P) in two steps: fluorination with aqueous 0.1 M kF at 20 °C for 24 h, then immersion in a mixed solution of NaNO_3_, Ca (H_2_PO_4_)_2_ H_2_O and H_2_O_2_ at 20 °C for 24 h; or - SrP one-step conversion (Mg-Sr–P) after immersion in a solution containing 0.1 M Sr (NO_3_)_2_ and 0.06 M NH_4_H_2_PO_4_ at 80 °C for 10 min, with pH being adjusted to 3.0 with diluting HNO_3_. Mg–Sr alloy.	CCK-8 assay at 1, 3 and 5 days. ALP staining assay at 7 days. Alizarin red S assay at 21 days. N = 3. Wilcoxon test (SAS 9.4). Type I error was set at 5%.	Higher cell proliferation for Sr–P coating and Ca–P coating i in comparison to Mg-Sr alloy and blank control. Severe degradation of Mg-Sr alloy with hydrogen release determined RMSCs adhesion reduction. Highest ALP activity and Alizarin red S for Sr-P coating.	[31]
Mouse, Osteoblastic cell line MC3T3-E1	AZ91-3Ca Mg alloy was coated through a hydrothermal process with a solution of 1M calcium nitrate (Ca(NO_3_)_2_ 4H_2_O) and 0.6 M diammonium hydrogen phosphate ((NH_4_)_2_HPO_4_) with pH 4, (CP4100) or pH 7 (CP7100) at 100 °C for 3 h. AZ91-3Ca Mg alloy	Alamar Blue assay at 1 and 3 days. SEM analysis at 3 days. RT-PCR analysis of Alp, Runx-2 and Col1A1 genes expression. N = 3. Unpaired two-tailed *t*-test. Type I error was set at 5%.	Higher cell proliferation for CaP-coated magnesium alloy in comparison to uncoated AZ391-3Ca alloy. Better adhesion on CaP coatings on AZ391-3Ca in comparison to control Higher osteogenic expression for cells exposed to CaP coatings in comparison to control alloy.	[32]
Rat, (4 weeks old male SPF SD rats) Bone marrow mesenchymal stem cells (BMSCs)	ZK60 magnesium alloy coated with HA: HA-ZK60. Sr-doped HA (3 concentrations: Sr 3%, Sr5% and Sr 10%). ZK60 Mg alloy.	Live/Dead assay at 24 h of incubation. Cell adhesion analysis at 12 h. RT-PCR analysis d at 3 days of for Alp, Bmp-2, Col1A-1, Ocn, Opn, Runx-2 genes ALP staining at 3 days. One-way ANOVA. Type I error was set at 5%.	Improved viability and cell adhesion for Sr-doped coatings in comparison to HA- ZK60 and ZK60 Mg alloy. Enhanced osteogenic differentiation for all Sr-doped HA coatings in comparison with HA-coated alloys.	[33]
Mouse, Osteoblastic cell line MC3T3-E1.	JDBM alloy with hydrofluoric acid: HF-JDBM. JDBM alloy coated with DPCD (brushite) precursor: DPCD-JDBM. JDBM alloy coated with hierarchically structured HA and were subjected to low-temperature hydrothermal treatment with varying time: 20 min (HA-20 min), 1 h (HA-1 h), 3 h (HA-3 h), 6 h (HA-6 h) and 12 h (HA-12 h): HA-JDBM. Mg–Nd–Zn–Zr alloy: JDBM	CCK-8 assay after 1, 3 days of culture. Cell adhesion, proliferation assay and microscopic analyses. SEM analysis at 1 day. ALP staining assay at 14 days. N = 3 Student’s *t*-test or one-way ANOVA (SPSS v.24). Type I error was set at 5%.	Significantly higher cell proliferation for DCPD and HA-1h than the control group. Higher viability for DCPD group in comparison to control samples. Better adhesion for DCPD, HA-1 h, HA-3 h and HA-12 h than JDBM samples. Higher ALP expression for control samples in comparison to HF-JDBM and DCPD while HA-1 h, HA-3 h and HA-12 h showed an enhanced osteogenic differentiation compared to JDBM samples.	[34]
Human Osteosarcoma cell lines MG63	AZ31 magnesium alloy coated, through electrospinning technique, with different percentage of Polycaprolactone (AZ31-PCL), pluronic F127 and nanohydroxyapatite (nHA): -AZ31-PCLPnHA (10%, 20% or 30%). -AZ31-PCL. -AZ31 Mg alloy.	MTT assay at 24 h and 72 h. Fluorescence microscopy images-acridine orange stained at 24 h. Cell morphology at 24 h. ALP staining assay at 7 and 14 days. Alizarin Red-S and Sirius Red staining at 7 and 14 days. RT-PCR analysis of osteogenic genes at 7 and 14 days. N = 3. Two-way ANOVA and Sidak’s multiple comparisons post hoc test. Type I error was set at 5%.	Higher viability of nanocomposite electrospun scaffolds at 72 h. Good viability with fluorescent staining for uncoated and nanocomposite-coated AZ31 materials. Increased adhesion characteristics on AZ31-PCLPnHA nanocomposite samples extract than AZ31, AZ31-PCL and control. Increasing ALP assay for all the samples proportionally to culture time. More mineralized calcium nodules for cells incubated with extracts of nanocomposite scaffolds showed than uncoated and PCL-alone-coated samples. Denser collagen secretion (Sirius red dye) for AZ31-coated nanocomposite than those with uncoated AZ31 and AZ31-PCL. Higher levels of ALP, Bmp-2 and Runx-2 for AZ31- PCLPnHA-coated sample extract than uncoated AZ31 sample extract	[35]
Mouse, Osteoblastic cell line MC3T3-E1	Titanium Alloy coated with High- and low-molecular-weight Hyaluronic Acid and HA substrates, respectively: h-HA-Ti and l-HA-Ti. AZ31 Mg alloy coated with corrosion-resistant silane: hHA-AZ31 and l-HA-AZ31. Titanium Alloy. AZ31 Mg alloy.	SEM after 24 h of cell culture. DNA Quantification was considered as a measure of cell adhesion and proliferation after 1, 3, 7 and 14 days of culture. ALP staining assay Total Collagen Content (through hydroxyproline assay kit) at 3, 7 and 14 days. SEM-EDX for Osteoblasts Mineralization at 14 days. N = 3 One-way ANOVA followed by post hoc Tukey test.	Greater number of adhered osteoblasts with spindle-shaped morphologies on Ti substrate control Significantly enhanced osteoblast cell proliferation (DNA quantification) for Ti alloy compared to other samples. Increased ALP expression for h-HA-AZ31 and h-HA-Ti in comparison to other samples. Higher Collagen Content for h-HA-AZ31 and h-HA-Ti surfaces than other samples. SEM images of mineralization showed an uniformly distributed Ca−P mineral particles on and around the MC3T3-E1 cells adherent to l-/h-HA-Ti and Ti only substrates.	[36]
Mouse, Osteoblastic cell line MC3T3-E1	Mg-BP/PLGA obtained through liquid exfoliation method for BP and PLGA coating derived from dip-coating method. Mg-BP/PLGA treated in solution with dexamethasone (0.4 mg/mL) for loading Dex to the BP nanosheets: Mg-Dex/BP/PLGA. Black phosphorus was prepared using a liquid exfoliation method: Mg-BP. Poly(D,L-lactide-co-glycolide): Mg-PLGA. ZX11 Mg alloy (Mg-1.0 wt %/Zn-1.0 wt %/Ca).	CCK8 assay and SEM analysis at 3 days. Alizarin Red staining by confocal microscopy at 7 days. ALP activity assay at 7 days. N = 3. *t*-test was performed using SigmaPlot 12.0 (Systat Software Inc., San Jose, CA, USA).	Slightly higher cytocompatibility for Mg-BP/PLGA in comparison to Mg-PLGA. No cells viability on Mg and Mg-BPC,. Cell adhesion was observed on Mg-PLGA and Mg-BP/PLGA substrates compared to Mg and Mg-BP. The Alizarin Red staining revealed an increase in mineralization nodules formations for Mg-Dex/BP/PLGA in comparison to Mg-BP/PLGA. ALP activity assay confirmed Alizarin Red staining	[37]
Mouse, Macrophage cell line RAW 264.7. Mouse, Osteoblastic cell line MC3T3-E1.	AZ31-LDH# obtained after putting AZ31 in a Teflon-lined stainless 50 mL of Al(NO_3_)_3_ after being reacted at 120 °C for 12 h. AZ31- LDH/PDA# obtained after immersion of AZ31-LDH in polydopamine (PDA) solution. Zn-(1#,2# and 3#) Alloy obtained after immersion of AZ31-LDH to dopamine solution plus different concentration of Zn(NO_3_)_2_ (1, 2 and 5 mg/mL). AZ31 Mg alloy.	Cell adhesion at 1, 4 and 24 h. Live/Dead staining at 7 day. ALP staining and RT-PCR analysis measured at 7 and 14 days on sample extracts Two-way ANOVA followed by the Tukey post hoc test (GraphPad). Type I error was set at 5%.	Higher spreading cell area for LDH/PDA# and Zn-2# samples than the cells in AZ31and LDH# samples. Good viability for all the three coated samples; no viable cells on the surface of AZ31 Mg alloy. Highest ALP activity for cells cultured in the extract of Zn-2# sample. No differences in gene expression between the cells cultured in all the four extracts for Runx-2 and Col1A1. Higher ALP, COL-I and OCN expression for cells cultured with extract of Zn-2 than AZ31.	[38]
Mouse, Osteoblastic cell line MC3T3-E1 and Mouse, Macrophage cell line RAW 264.7	AZ31 magnesium alloy coated with HA: AZ31-HA. AZ31 magnesium alloy coated with HA and chitosan–metformin (CS-MF): AZ31/HA/CS-MF. AZ31 Mg alloy.	Live/Dead staining at 24 h. Cell adhesion and morphology at 1 and 3 days. RT-PCR analysis at 7 and 14 days ALP staining at 7 and 14 days One-way or two-way ANOVA with Bonferroni post hoc tests.	No significant difference in the cell survival rate in the AZ31/HA/CS-MF and MF (drug) groups; cell survival rate of the AZ31 and AZ31/HA was meaningfully lower than that of the blank group. Good adhesion on the surface of AZ31/HA and AZ31/HA/CS-MF. Significant increase expression of osteogenic gene in the AZ31/HA/CS-MF extract than AZ31 and AZ31/HA. Increased ALP activity for AZ31/HA and AZ31/HA/CS-MF compared to other samples.	[39]
Mouse, Macrophage cell line RAW 264.7. Human, Bone marrow mesenchymal stem cells (BMSCs).	WE 43 Mg with 3-layered coatings: - inner layer of MgO; - interlayer of poly-L-lactide containing curcumin-loaded F-encapsulated mesoporous silica nanocontainers at 0, 5, 10 and 20 wt% concentration (0FMSN, 5FMSN, 10FMSN, 20FMSN); - outer layer of dicalcium phosphate dehydrate.	Live/Dead at 1, 3 and 5 days. Conditioned culture medium (CCM) was prepared from 3-days culture of Raw 64.7 cells seeded on each F-coated Mg discs. On BMSCs cultured with CCM: RT-PCR analysis at 7 and 14 days to test expression of: Runx2, Alp, Opn and Ocn. ALP staining assay at 7 days. Alizarin Red staining at 14 and 21 days. N = 3. One-way ANOVA followed by a least-significant difference post hoc test was performed. Type I error was set at 5%.	No significant differences in viability for Raw cells on the coated Mg disc Higher gene expression, ALP and Alizarin Red staining for BMSCs cultured with cFMSN-containing coatings than 0FMSN. Greater immunomodulatory efficiency for 20FMSN 10FMSN and 5FMSN.	[40]
Mouse, Macrophage cell line RAW 264.7.	AZ31 magnesium alloy coated with poly(ε-caprolactone) (PCL) and functionalized with coumarin (CM) and zinc oxide nanoparticles (ZnO): -Mg-PCL-CM-ZnO. -Mg-PCL-ZnO. -Mg-PCL. -Mg-PCL-CM. AZ31 Mg alloy.	Live/Dead staining at 1and 3 days Osteoclast Differentiation Assay: TRAP analysis at 7 days. N = 3. One-way or two-way ANOVA with Tukey’s multiple comparisons test. Type I error was set at 5%.	CCK-8 assay revealed a time-dependent increase in the number of metabolically active viable cells after incubations with alloys. Osteoclasts increased in the following order: TCPS(-) negative control < Mg -PCL-CM-ZnO < Mg-PCL-ZnO < Mg < Mg-PCL < Mg-PCL-CM < RANKL.	[41]
Mouse, Osteoblastic cell line MC3T3-E1	Mg alloy WE43 coated with Poly(ether imide): PEI-coated Mg alloy. Mg alloy coated with Tantalum/poly(ether imide): Ta/PEI-coated Mg alloy. WE43 Mg alloy.	Live-cell staining assay at 6 and 24 h of culture. CyQUANT cell proliferation assay kit to investigate the amount of DNA 24 h, 3 and 5 days. ALP staining assay at 10 days. N = 3. One-way ANOVA with Tukey post hoc comparison. Type I error was set at 5%.	Cell proliferation increased during the experimental times. Significantly increased of DNA content for the PEI-coated and Ta/PEI-coated Mg surfaces during the time, No osteogenic ability for bare Mg; increase ALP activity for e PEI-coated Mg and for Ta/PEI-coated Mg in comparison to bare and PEI-coated Mg alloy.	[42]
Mouse, Osteoblastic cell line MC3T3-E1	MgA alloy coated with hydrogel micropatterns of poly(alendronate sodium methacrylate)/poly(dimethyldiallylammonium chloride)/poly(ethylene glycol) diacrylate (PALNMA/PDADMAC/PEGDA) named: MgA-Mg(OH)_2_- PALNMA/PDADMAC/PEGDA. MgA-Mg(OH)_2_- PALNMA/PDADMAC/PEGDA-HA/BBR obtained combining hydrothermal treatment, patterning and layer-by-layer assembly technology. Mg(OH)_2_ obtained by using solvothermal method. AZ31 Mg alloy (MgA)	MTT assay at 1 day and 3 ALP staining assay at 3 days. Alizarin Red staining at 14 days of treatments. N = 3. Student *t*-tests. Type I error was set at 1%.	Higher cell viability for MgA-Mg(OH)_2_-PALNMA/PDADMAC/PEGDA-A/BBR than the MgA, ALP and Alizarin Red assays confirmed the viability results	[43]
Mouse, Osteoblastic cell line MC3T3-E1.	Mg-1Ca-0.2Mn-0.6Zr alloy coated with cellulose acetate (CA) coatings: Mg-CA. Mg-1Ca-0.2Mn-0.6Zr alloy CA coatings functionalized with resveratrol (Res): Mg-CA-Res.	Cell proliferation assessment (MTT) at 1, 3 and 5 days. ALP staining assay at 7 and 14 days. ECM analysis at 4 and 6 weeks. N = 3 ANOVA followed by Bonferroni multiple comparison test. Type I error was set at 5%.	Increased for Mg-CA-Res than control samples. Higher mineralization nodules formation for Mg-CA-Res extraction medium.	[44]

**Abbreviations:** wt%:weight percentage; Mg: Magnesium; Ge: Germanium; ALP: alkaline phosphatase; Al: Aluminum; Zr: Zirconium; DMEM: Dulbecco’s Modified Eagle Medium; FBS: Fetal Bovine Serum; N: Nitrogen; MTT: 3-(4,5-dimethylthiazol-2-yl)-2,5-diphenyl-2H-tetrazolium bromide; Ag: Silver; Gd: gadolinium; MAO: Micro-arc oxidation; SEM: Scanning Electron Microscopy; CCK-8: Cell Counting Kit-8; RT-PCR: reverse transcriptase-polymerase chain reaction; Runx-2: Runt-related transcription factor 2; Col1A1: Collagen type I alpha; Ocn: osteocalcin; Sr: Strontium; Ca: Calcium; PED: pulse electrodeposition; P: Phosphate; SrP: strontium phosphateBmp-2: Bone morphogenetic protein-2; Mn: Manganese; Fe: Iron; PEO: plasma electrolytic oxidation; Zn: Zinc; ECM: Extracellular matrix mineralization; HT: hydrothermal treatment; Bsp: Bone sialoprotein; Opn: Osteopontin; Ocn: Osteocalcin; CLSM: Confocal Laser Scanning Microscope; FHA: fluoridated hydroxyapatite; PIII: plasma immersion ion implantation; HA: hydroxyapatite; Ta: tantalum; LDH: Layered Double Hydroxides; DPCD: brushite precursor; PCL: Poly(ε-caprolactone); BP: Black Phosphorus; PLGA: Poly(d,l-lactide-co-glycolide); HF: hydrofluoric acid; nHA: nanohydroxyapatite; Dex: dexamethasone; PDA: Polydopamine; CS-MF: chitosan–metformin; FMSN: F-encapsulated mesoporous silica nanocontainers; CCM: Conditioned Culture Medium; CM: Coumarin; ZnO: zinc oxide nanoparticles; PEI: Poly(ether imide); CA: Cellulose Acetate; Res: resveratrol.

**Table 2:** Selected in vivo studies included in the systematic review.

Aim	Species, Strain, Sex, Age, Number	Model and Site	Mg Alloy Controls	Follow-Up and Evaluations’ Statistical Analysis	Main Results	Ref.
Evaluation of in vivo degradation behavior and osseointegration of open-pored scaffolds made of the two magnesium alloys LAE442 ((Mg with 4% Li, 4% Al, 2% rare earths), n = 40) and La2 (magnesium with 2% lanthanum n = 40).	Rabbits, Zika (Asamhof, Kissing, Germany), mature female, n = 60.	Cylindrical scaffolds (Ø 4 mm, length 5 mm) with pores (max. 500 µm, porosity 41.4%) inserted in 6 mm deep hole into the cancellous part of the greater trochanter in the direction of the femoral head (4 mm drill).	TCP (porous ß-tricalcium phosphate) scaffolds, n = 40.	Radiology and Micro-CT (micro-computed tomography) at 6, 12, 24 and 36 weeks after surgery. The statistical evaluation of the data was carried out using SPSS Statistics 25.0. Not normally distributed data analyzed by Kruskal–Wallis test. Pair-wise comparisons by adjusting *p*-value according Bonferroni correction. Type I error was set at 5%	The open-pored scaffolds LAE442 and La2 showed no clinical complications. La2 scaffolds showed a relatively fast, inhomogeneous degradation with an inferior osseointegration compared to the LAE442 scaffolds. LAE442 scaffolds showed (1) a very slow, homogeneous degradation, maintaining their original form until the end of the investigation period; (2) a better osseointegration which made them more suitable for examination in weight-bearing bone defects.	[45]
Evaluate and quantified the degradation and osseointegration behavior of two biodegradable Mg alloys based on gadolinium (Mg–10Gd, (n = 30) Mg with Gd at 10 wt.% and Mg–5Gd, (n = 30) Mg with Gd at 5wt.%).	Rats, Sprague Dawley, adult male, average b.w. 350 g, n = 60.	A 1.6 mm diameter hole in both tibia metaphyses. Each rat received either 2 Mg-based monocortical screws (one Mg–10Gd and one Mg–5Gd) or two non-Mg monocortical screws (PEEK and Ti), with random allocation to the left and right leg.	PEEK (polyether ether ketone, n = 30) and Ti: (Titanium, n = 30) screws.	Retrieved bone implant sites were analyzed after 4, 8 and 12 weeks (20 rats per time). SRμC (Synchrotron radiation based micro computed tomography). Histology Assays with Toluidine Blue-Pyronine Y and TRAP staining. Two-way ANOVA (material, time point) test followed by multiple comparisons with Bonferroni adjustment of *p*-values (MATLAB R2019b and SPSS v.26). Type I error was set at 5%.	TRAP staining showed the highest osteoclast activation for all materials at 4 weeks, with a significant difference in comparison to the other experimental time. Samples of Ti decrease osteoclast activation gradually over time. Mg-5Gd, Mg-10Gd and PEEK samples showed a decrease from 4 to 8 weeks, then slightly increase between 8 and 12 weeks, but without statistically significant. The Mg-5Gd slides showed significantly higher TRAP staining at 4 weeks, compared to the other materials. The differences among Mg-10Gd, PEEK and Ti were not statistically significant at any of the three time points. Bone tissue surrounded the implants of all materials already at 4 weeks, and its amount increased up to week 12, extending even in those areas that initially were not in contact with bone surfaces. A higher amount of woven bone was observed around the Mg alloys, at 4 weeks, while the bone around Ti and PEEK looked more mature. Newly formed bone was found predominantly facing regions of the implants that were more degraded. The degradation rate of Mg-5Gd was faster and less homogeneously than Mg-10Gd.Both alloys gradually form a stable degradation layer at the interface and were surrounded by new bone tissue.	[8]
Evaluation of the performances of novel Mg-Ge based biodegradable metals (n = 18).	Rabbits New Zealand, female, b.w. 2–2.5 kg, n = 12.	The screw was implanted in pre-drilled hole of 2.2 mm in diameter on lower edge of the lateral tibial plateau.	Absence of implant as control (3 animals).	Postoperative observation, Micro-CT and Histology (H&E or toluidine blue staining of undecalcified sections) at 1, 2, 3 months from surgery Statistical analysis was performed with SPSS 18.0 software. One-way analysis of variance (ANOVA) followed by Tukey test.	Mg-Ge based biodegradable implant showed a good in vivo degradation rate (0.6 mm/y). This implant had a limited H_2_ generation during implant degradation without bone destruction. It potentially completed the absorption in 3 months. It presented a good osseointegration proved by new bone, that grow directly onto the implant.	[16]
Evaluation of the mechanical quality of the bone–implant interface and the amount of peri-implant bone of a biodegradable magnesium alloy (Mg-Y-Nd-HRE) pin (n = 62).	Rats, Sprague-Dawley, 5-week-old male, b.w. 120–140 g. n = 72 animal.	Cylindrical implant inserted in transcortical drill into the mid-diaphysis of each femoral bone.	Self-reinforced PLGA implant (n = 62 implants, n = 26 at 4 weeks, n = 26 at 12 weeks, n = 20 at 24 weeks).	Biomechanical tests, Micro-CT, Histology (Undecalcified sections stained with Lévai–Laczkó procedure), SEM and Interleukin-6 (IL-6) assay and standard differential white cell counts at 4 (n = 13 animal for implant type), 12 (n = 13 animal for implant type) and 24 (n = 10 animal) weeks. Data were analyzed by the statistical software PASW Statistics 18.0 (SPSS Inc, Chicago, IL, USA). Differences between the implant types after each implantation period were analyzed by Mann–Whitney U test. Differences between different implantation periods within each implant type were analyzed by Kruskal–Wallis tests and subsequent by pairwise analysis by Mann–Whitney U tests. Significance level was adjusted for multiple testing by Bonferroni correction. Type I error was set at 5%.	Push-out testing revealed highly significantly greater shear strength (τu) in biodegradable magnesium alloy implant respect to PLGA control in all implantation periods. τu significantly increased between 4 and 12 weeks in the two implant types (Mg-alloy 2.54-fold and PLGA implants 2.65-fold), while no further significant increase was observed between 12 and 24 weeks. Histologically, the biodegradable Mg-alloy implant presented a significantly greater BV/TV respect to copolymeric control at 4 weeks. At 12 and 24 weeks, no significant differences in BV/TV were found between the two degradable implant types. Undecalcified thin ground sections parallel to the long axis of the implants revealed direct apposition of bone to the implant surface for both implant types. No fibrous capsule formation or inflammatory foreign-body reaction around the implant surface were found. Within the cortical portions of the femur, signs of osteonal bone remodeling were detectable, as circular resorption cavities, which were present in a higher number in the Mg-alloy group. There was no significant difference in IL-6 serum levels between rats which received Mg-alloy rods and those implanted with PLGA rods after implantation.	[46]
Study feasibility of as-cast β-phased Mg-30 wt% Sc (Scandium) alloy (n = 36 implants) as biodegradable orthopedic implant.	Rats, Sprague-Dawley, male, b.w. 200–250 g, n = 36.	A cylindrical scaffold inserted in a bone defect (1 mm diameter and 6.5 mm deep) at the lateral femur epicondyle on both femurs.	HP-Mg (High Pure Magnesium 99.99 wt%) (n = 36 implants)	In vivo micro-CT analysis at 2 weeks and ex vivo micro-CT at 4 (n = 6 animal for implant type), 12 (n = 6 animal for implant type) and 24 (n = 6 animal for implant type) weeks. Histological analysis at 4 weeks with H&E staining. Hematology analysis at 4, 12 and 24 weeks. Characterization of corrosion products at 4- and 12 weeks post-operation. One-way ANOVA followed by Tukey test (SPSS v.20). Type I error was set at 5%.	β phased Mg-30 wt%Sc alloy group showed 1) no gap between implant and bone. at 2 weeks after implantation; 2) a degradation rate of 13 ± 3% after 24 weeks implantation with little gas generation at initial stage HP-Mg group degraded rapidly (totally degraded after 2 weeks) and the bone remodeling was disturbed by gas released. No pathological changes or tissue necrosis were exhibited in both groups. No abnormalities on blood biochemistry or trace elements detection were found.	[47]
Evaluation of the degradation behavior of PEO surface-modified ZX00 (Mg–0.45Zn–0.45Ca, in wt%) screws (n = 36 implants).	Miniature Pigs, Göttingen, average b.w. 50.61 kg (± 30 kg), n = 13 castrated males and n = 5 females (group 6 months: 1 female/5 male, group 12 months: 1 female/5 male, group 18 months: 3 female/2 male)	The frontal bone was chosen because of its comparability to the midfacial area of humans. Four screws, two of each type, were randomly implanted in every pig in a circle with an app. 20 mm distance between screws.	ZX00MEO screw (n = 36 implants).	At 6, 12 and 18 months from surgery: Percentage of the bone surrounding the devices was measured in 3D by micro-CT. Percentage of residual screw fractions in bone was calculated in 3D as volume %SV/TV and in 2D as area %SA/TA. Measurement of bone implant contact (%BIC). Histology Giemsa-stained sections for qualitative assessment of new bone formation and structure. STATA 15 software was used to analyze data. Separate linear mixed regression models were used. Results were expressed as regression coefficients with standard errors and 95% confidence intervals. Type I error was set at 5%.	Radiological 3D evaluation showed a significantly higher residual device volume (%SV/TV) for the PEO-modified group after 12 and 18 months implantation period in the frontal bone of the minipigs. The quantification of the surrounding bone also revealed a significantly higher quantity of bone around the ZX00MEO-PEO samples after 18 months. ZX00MEO alloy showed beneficial properties as bioabsorbable implant material and proved safety and performance on device level. PEO-surface modification shows to improve the bioabsorption behavior of ZX00MEO alloy by reducing degradation rates.	[48]
Evaluation of long-term implant degradation behavior, quantity and quality of the surrounding bony tissue after implantation of WE43-PEO alloy (n = 8 implants).	Miniature Pigs, Göttingen, skeletally mature females and castrated males, b.w. 53.4 ± 12.9 kg n = 8 (2 females; 6 males).	Periosteum were mobilized and monocortical screws were inserted to fixate the plate within the diaphyseal area of the bone (tibias OR femurs)	WE43 implants (n = 8 implants).	Micro-CT and histological analysis (Giemsa stained undecalcified sections) at 6 and 12 months from surgery. Prism 8.1.1 software was used for statistical analysis. Mann–Whitney U test was applied for the comparison of averages between two groups and Kruskal–Wallis tests was applied for the comparison of averages between more than two groups, respectively. Type I error was set at 5%.	PEO slowed down the degradation behavior of WE43 magnesium implants in vivo. Surface modification resulted in an increased residual screw volume and improved osseointegration after 6 months. PEO was associated with a moderate osteostimulating effect with respect to the bone marrow and an increased formation of new bone with respect to the subperiosteal region until 12 months from surgery. Sufficient biocompatibility of surface-modified magnesium implants after six and twelve months in vivo.	[49]
Evaluation of the immersion processes to construct Mn and Fe oxyhydroxide duplex layers on the PEO-treated AZ31 (PEO–Mn/Fe) to solve the problem of pores and cracks that easily formed on the PEO surface, which compromise the resistance of corrosion of AZ31 implant obtained with PEO method.	Rats, Sprague–Dawley, male, b.w. 250–300 g, n = 12 divided in four groups (n = 3): Control (n = 6 implants), PEO-Fe (n = 6 implants); PEO-Mn (n = 6 implants); and PEO-Mn/Fe (n = 6 implants).	Cylindrical scaffolds (2 mm in diameter and 8 mm in length) were implanted in the trochlear groove of the femur reaching the marrow cavity.	PEO AZ31 implant (control, n = 6 implants).	Micro-CT at 8 weeks after surgery. Histological analysis on undecalcified sections stained with van Gieson’s solution. Two-way ANOVA followed by Tukey’s post hoc test using the GraphPad Prism 8.3.0. Type I error was set at 5%.	The micro-CT results suggested that the structures of all femurs were normal, and no bone resorption or osteonecrosis was present. Histology showed that the greatest amount of newly formed bone was observed in the PEO-Mn/Fe group, suggesting that it had the best osteogenesis performance due to its resistance to corrosion and its prolonged release of Mg, Fe and Mn ions. These implants showed an improved bone regeneration in vivo.	[22]
Evaluate the degradation behavior, osseointegration and gas release of two types of open porous LAE442 scaffolds (P1: 400 µm; P2: 500 µm) with two distinct layers of coating, first the base layer of MgF2 and furthermore an additional layer of either PLA (PLA-P1, n = 32 implants; PLA-P2 n = 32 implants) or CaP (CaP-P1, n = 32 implants; CaP-P2, n = 32 implants).	Rabbits, ZiKa (Asamhof, Kissing, Germane), >6-months old female, b.w. 3.96 ± 0.27 kg n = 80 divided in 5 groups of n = 16 animals each.	Cylindrical scaffolds (Ø 4 mm, length 5 mm) with interconnecting pores (max size 400 µm or 500 µm) in a 6 mm deep hole was drilled into the trochanter with a 4 mm drill.	TCP scaffolds (n = 32 implants).	X-ray and micro-CT assessments were performed directly post- surgery, every 2 weeks until week 12 and then every 4 weeks until the end of the observation period (36 weeks) to evaluate scaffold degradation and osteointegration. Statistical analyses were performed with SPSS Statistics 25. Kruskal–Wallis test or ANOVA followed by post hoc test with a Bonferroni correction. If only two samples had to be compared, a Mann–Whitney U test was used instead. Type I error was set at 5%.	All types of LAE442 scaffolds showed a much slower degradation than TCP, which degraded strongly from week 6 due to its low degradation resistance. MgF2 with CaP or PLA in LAE442 scaffolds were able to reduce the degradation rate and gas formation (only MgF_2_–CaP coating) of Mg implants compared to Mg implants coated with only MgF_2_. Additional surface coatings of LAE442 alloys support their ability for osseointegration and positively influence the degradation behavior. CaP coating proved to be even more promising in terms of osseointegration than the PLA coating. Scaffolds with PLA coating, however, degraded more slowly despite increased gas formation.	[50]
Evaluate corrosion resistance and bone formation ability of the Zr-N-implanted AZ91 (Mg with Al 9 wt.% and Zn 1 wt.%) Mg alloy (n = 8 implants).	Rats, Sprague-Dawley, 33-month-old female n = 8.	A cylindrical scaffold inserted in a bone defect (2 mm diameter and 6 mm deep) at the lateral femur epicondyle on both femurs.	Unimplanted AZ91 rods (n = 8 implants).	Micro-CT assessments at 0, 1-, 4-, 8- and 12 weeks post-surgery. Histological evaluation after 12 weeks with H&E staining (decalcified sections) and Van Gieson’s picrofuchsin (undecalcified sections) staining. Sequential fluorescence labeling at 3 (tetracycline hydrochloride), 6 (alizarin red) and 9 (calcein) weeks after surgery to assess new bone formation. Student’s *t*-test. Type I error was set at 5%.	Histological evaluation showed a more intact and mature bone tissue around Zr-N-implanted AZ91 implant in comparison with AZ91 implant. The BIC value for the Zr-N-implanted AZ91 implants was significantly higher respect AZ91 groups. Around the Zr-N-implanted AZ91 implant, more density and greater areas of tetracycline hydrochloride fluorescence, alizarin red S fluorescence and calcein fluorescence (newly formed bone tissues) were observed respect to AZ91 implant.	[17]
Evaluate bone regeneration and material degradation and the feasibility of the MgF2-coated AZ31 (FAZ31) scaffold (n = 30 implants).	Rabbits, New Zealand b.w. 2.5–3.0 kg n = 30: n = 15 AZ31 scaffold group; n = 15 FAZ31 scaffold group.	A porous cylindrical implant inserted in a bone defect (5 mm diameter and 4 mm deep) at the lateral femur epicondyle on both femurs.	AZ31 (magnesium 3 wt. % Al, 1 wt. % Zn) scaffold (n = 30 implants).	Micro-CT assessments at 6, 12- and 18 weeks post-surgery. Histological observation through H&E and immunohistochemistry (IHC) staining (decalcified sections) and with fluorescent labeling (calcein, alizarin red and tetracycline hydrochloride at 3, 9 and 15 weeks, respectively) and Van Gieson’s picrofuchsin staining (undecalcified sections) to identify new bone formation. One-way ANOVA followed by Student–Newman–Keuls post hoc tests. Type I error was set at 5%.	FAZ31 scaffolds exhibited enhanced corrosion resistance and improved cytocompatibility compared to the bare AZ31 scaffolds. FAZ31scaffolds showed enhanced osteogenic activity compared to the AZ31 scaffolds. Superior osteoconductive and osteoinductive properties of FAZ31 scaffolds compared to the AZ31 scaffolds bare.	[25]
Evaluation of corrosion resistance and bone formation ability of the functionalized TiO_2_/Mg_2_TiO_4_ nanolayer constructed on the surface of WE43 (magnesium with 3.5 wt.% Yttrium, 2.3 wt.% Neodymium, 0.5 wt.% Zirconium) implant by using plasma ion immersion implantation (PIII) technique (n = 20 implants).	Rats, Sprague-Dawley, 13-week-old female, b.w.: 300–350 g, n = 30.	A cylindrical scaffold inserted in a bone defect (2 mm in diameter; 6 mm in depth) on the end of lateral epicondyle on the right and left femur of rats.	Titanium (positive control) (n = 20 implants) and untreated WE43 rods (n = 20 implants).	Micro-CT Histological analysis (undecalcified sections stained with Giemsa solution) Mechanical properties (push-out test of newly formed bone at eight and twelve weeks). at 1, 2, 4, 8 and 12 weeks. One-way analysis of variance using the SPSS software.	The multifunctional titanium oxide-based nanolayer hampered rapid corrosion of WE43 Mg alloy and promotes in-situ bone regeneration. New bone volume adjacent to PIII treated WE43 implants showed 175% growth, respect to the 97% of the Ti control and 28% of the untreated WE43 group, after 12 weeks post-surgery. The trabecular thickness and bone mineral density of surface treated group are also significantly higher than that of Ti and untreated WE43 groups. Newly formed bony tissue in surface treated group exhibits well mineralized structure and its mechanical property are almost restored to the level of surrounding mature bone.	[28]
Evaluation of corrosion resistance and osseointegration of WE43 Mg-based implants coated with 3-layered coatings: - inner layer of MgO; - interlayer of poly-L-lactide containing curcumin-loaded F-encapsulated mesoporous silica nanocontainers at 0, 5, 10 and 20 wt% concentration (0FMSN, n = 6 implants; - 5FMSN n = 6 implants; - 10FMSN, n = 6 implants; - 20FMSN n = 6 implants;); - outer layer of dicalcium phosphate dehydrate	Rats, adult b.w. about 250 g n = 18. (n = 6 femoral site for each tested implants).	Cylindrical scaffold inserted in a hole (∅ 1.6 mm) in the right and left femoral condyles of each rat.	WE43 Mg implant n = 6 implants.	Micro-CT to evaluate the remaining volumes of the coated and bare Mg implants and peri-implant bone formation at 4 weeks after surgery. Histological examinations assays on undecalcified sections stained with Van Gieson’s picric fuchsine at 4 weeks after surgery. The data were analyzed using SPSS 16.0 software (USA). A one-way ANOVA followed by a least-significant-difference (LSD) post hoc test was used. Type I error was set at 5 and 1%.	The cFMSNs coatings exhibited self-healing effects and inhibit sensibly the corrosion on Mg substrates. The largest amount of cFMSNs contained coating (20FMSN) showed the most effective protection to Mg substrate against corrosion. cFMSNs coatings induced a quick phenotype switch of the macrophages from M1 to M2, and then from pro-inflammatory to anti-inflammatory activities. 20FMSN modulated its microenvironment more efficiently (anti-inflammation, osteodifferentiation and ECM mineralization, enhanced osseointegration) compared to the coatings 10FMSN and 5FMSN.	[40]

**Abbreviations**: Li: Lithium; Al: Aluminum; La: Lanthanum; TCP: ß-tricalcium phosphate; Micro-CT: Micro-computed tomography; Gd: Gadolinium; PEEK: polyetheretherketone; Ti: Titanium; SRμCT: Synchrotron radiation based micro computed tomography; TRAP: tartrate-resistant acid phosphatase; Ge: Germanium; H&E: hematoxylin–eosin; H2: Hydrogen gas; Y; Yttrium; Nd: Neodymium; PLGA: poly (l-lactic-co-glycolic acid); SEM: Scanning Electron Microscopy; IL-6: Interleukin-6; BV/TV: interface-near bone volume per tissue volume;; Sc: Scandium; HP: High Pure; PEO: plasma-electrolytic oxidation; Zn: Zinc; Ca: Calcium; %SV/TV: percentage of Surface Volume/Total Volume; %SA/TA: percentage of Surface Area/Total Area; BIC: bone–implant contact; Mn: Manganese: Fe: Iron; PLA: Polylactic acid; Zr: Zirconium; N: Nitrogen; IHC: immunohistochemistry; PIII: plasma ion immersion implantation technique; cFMSNs: curcumin-loaded F-encapsulated mesoporous silica nanocontainers.

## Data Availability

All data are available in the manuscript and in the Supplementary Files.

## Supplementary Materials

The following supporting information can be downloaded at: https://www.mdpi.com/article/10.3390/ijms25010282/s1.

## Abbreviation

Mg: Magnesium; PCR: Polymerase Chain Reaction; ELISA: Enzyme Linked Immunosorbent Assay; Rob: Risk of Bias; OHAT: Office of Health Assessment and Translation; SYRCLE: SYstematic Review Centre for Laboratory animal Experimentation; ARRIVE: Animal Research: Reporting of In Vivo Experiments; Ti: Titanium; Ge: Germanium; Al: Aluminum; Zr: Zirconium; Ag: Silver; Gd: gadolinium; SEM: Scanning Electron Microscopy; Col1A1: Collagen type I alpha; Ocn: Osteocalcin; Sr: Strontium; Ca: Calcium; P: Phosphate; SrP: Strontium Phosphate; Mn: Manganese; Fe: Iron; Zn: Zinc; Bsp: Bone sialoprotein; Opn: Osteopontin; FHA: fluoridated hydroxyapatite; HA: Hydroxyapatite; Ta: Tantalum; HF: Hydrofluoric acid; nHA: nanohydroxyapatite; Li: Lithium; TCP: ß-tricalcium phosphate; Micro-CT: Micro-computed tomography; PEEK: Polyetheretherketone; MAO: Micro-Arc Oxidation; PED: Pulse Electrodeposition; PEO: Plasma Electrolytic Oxidation; HT: Hydrothermal Treatment; PIII: Plasma Immersion Ion Implantation.
