# Supplementary material for: Magnesium Alloys in Orthopedics: A Systematic Review on Approaches, Coatings and Strategies to Improve Biocompatibility, Osteogenic Properties and Osteointegration Capabilities
Source: Int J Mol Sci. 2023 Dec 24;25(1):282. doi: 10.3390/ijms25010282 (PMC10778661; doi:10.3390/ijms25010282)
Supplement: Supplementary file 1 [file ijms-25-00282-s001.zip › Supplementary File A.pdf]

Magnesium Alloys in Orthopedics: A Systematic Review on Approaches, Coatings and Strategies to improve Biocompatibility, Osteogenic Properties and Osteointegration Capabilities

Gianluca Giavaresi[a], Daniele Bellavia[a], Angela De Luca[a], Viviana Costa[a], Lavinia Raimondi[a], Aurora Cordaro[a], Maria Sartori[a], Silvio Terrando[b], Angelo Toscano[b], Giovanni Pignatti[b], and Milena Fini[c]*

Details of the search strategies performed in December 2022.

1 - Database: Medline (Pubmed)
Search: **((magnesium[MeSH Terms]) AND (alloy*[MeSH Terms])) AND (osseointegration OR osteointegration[MeSH Terms])** Filters: **English, from 2012 - 2022** Sort by: **Most Recent**

2 - Database: Web of Science
Search: TS=(magnesium AND alloy* AND (osseointegration OR osteointegration)) AND PY=2012:2022 and **Article** (Document Types) and **English** (Languages)

3- Database: EMBASE
Search: 'magnesium'/exp AND alloy* AND ('osseointegration'/exp OR 'osteointegration'/exp) AND (2016:py OR 2017:py OR 2018:py OR 2019:py OR 2020:py OR 2021:py OR 2022:py) AND 'article'/it AND [english]/lim

4-Database: Science Direct
Search: magnesium alloy osseointegration
2012-2022 – research article
