# Supplementary material for: Magnesium Alloys in Orthopedics: A Systematic Review on Approaches, Coatings and Strategies to Improve Biocompatibility, Osteogenic Properties and Osteointegration Capabilities
Source: Int J Mol Sci. 2023 Dec 24;25(1):282. doi: 10.3390/ijms25010282 (PMC10778661; doi:10.3390/ijms25010282)
Supplement: Supplementary file 1 [file ijms-25-00282-s001.zip › Supplementary File C.pdf]

Magnesium Alloys in Orthopedics: A Systematic Review on Approaches, Coatings and Strategies to improve Biocompatibility, Osteogenic Properties and Osteointegration Capabilities

Gianluca Giavaresi[a], Daniele Bellavia[a], Angela De Luca[a], Viviana Costa[a], Lavinia Raimondi[a], Aurora Cordaro[a], Maria Sartori[a], Silvio Terrando[b], Angelo Toscano[b], Giovanni Pignatti[b], and Milena Fini[c]*

Table 1. List of items extracted from studies.

- Study identification information;
- Funding source;
- Author conflicts of interest;
- Type of Study (i.e., in vitro and/or in vivo study, clinical study);
- Exposure (i.e., Type of magnesium alloy/controls, Duration of exposure; etc.);
- Population (i.e., Animal species, strain, sex, origin, age; Cell line, cell type or tissue; Source of cells or tissue culture, etc.);
- Dosing methodology (i.e., Performed Tests, Treatment period, Replicates etc.);
- Controls (positive, negative, vehicle);
- Randomization procedure, allocation concealment, blinding procedure during outcome assessment(s);
- Number of replicates per group
- Main outcome;
- Statistical methods (e.g., statistical test used, significance levels).
